# NK cell-triggered CCL5/IFNγ-CXCL9/10 axis underlies the clinical efficacy of neoadjuvant anti-HER2 antibodies in breast cancer

**DOI:** 10.1186/s13046-023-02918-4

**Published:** 2024-01-03

**Authors:** Sara Santana-Hernández, Jesús Suarez-Olmos, Sonia Servitja, Pau Berenguer-Molins, Marcel Costa-Garcia, Laura Comerma, Anna Rea, Julia Perera-Bel, Silvia Menendez, Oriol Arpí, Begoña Bermejo, María Teresa Martínez, Juan Miguel Cejalvo, Iñaki Comino-Méndez, Javier Pascual, Emilio Alba, Miguel López-Botet, Federico Rojo, Ana Rovira, Joan Albanell, Aura Muntasell

**Affiliations:** 1 Hospital del Mar Medical Research Institute (IMIM), Barcelona, Spain; 2 Oncology Department, Hospital del Mar, Barcelona, Spain; 3 Centro de Investigación Biomédica en Red de Cáncer (CIBERonc), Madrid, Spain; 4 Pathology Department, Hospital del Mar, Barcelona, Spain; 5 University Pompeu Fabra, Barcelona, Spain; 6 Department of Oncology, Hospital Clínico de Valencia, Valencia, Spain; 7 Hospitales Universitarios Regional y Virgen de La Victoria, Málaga, Spain; 8 The Biomedical Research Institute of Málaga, Málaga, Spain; 9 Department of Pathology, IIS ‘Fundación Jimenez Díaz University Hospital’, Madrid, Spain; 10 Universitat Autònoma de Barcelona, Hospital del Mar Research Institute (IMIM), Doctor Aiguader, 88, 08003 Barcelona, Spain

**Keywords:** NK cells, Anti-HER2 antibodies, Biomarker, HER2 positive breast cancer, CCL5, IFN-ɣ, CXCL9

## Abstract

**Background:**

The variability in responses to neoadjuvant treatment with anti-HER2 antibodies prompts to personalized clinical management and the development of innovative treatment strategies. Tumor-infiltrating Natural Killer (TI-NK) cells can predict the efficacy of HER2-targeted antibodies independently from clinicopathological factors in primary HER2-positive breast cancer patients. Understanding the mechanism/s underlying this association would contribute to optimizing patient stratification and provide the rationale for combinatorial approaches with immunotherapy.

**Methods:**

We sought to uncover processes enriched in NK cell-infiltrated tumors as compared to NK cell-desert tumors by microarray analysis. Findings were validated in clinical trial-derived transcriptomic data. In vitro and in vivo preclinical models were used for mechanistic studies. Findings were analysed in clinical samples (tumor and serum) from breast cancer patients.

**Results:**

NK cell-infiltrated tumors were enriched in *CCL5/IFNG-CXCL9/10 *transcripts. In multivariate logistic regression analysis, *IFNG* levels underlie the association between TI-NK cells and pathological complete response to neoadjuvant treatment with trastuzumab. Mechanistically, the production of IFN-ɣ by CD16^+^ NK cells triggered the secretion of CXCL9/10 from cancer cells. This effect was associated to tumor growth control and the conversion of CD16 into CD16^-^CD103^+^ NK cells in humanized in vivo models. In human breast tumors, the CD16 and CD103 markers identified lineage-related NK cell subpopulations capable of producing CCL5 and IFN-ɣ, which correlated with tissue-resident CD8^+^ T cells. Finally, an early increase in serum CCL5/CXCL9 levels identified patients with NK cell-rich tumors showing good responses to anti-HER2 antibody-based neoadjuvant treatment.

**Conclusions:**

This study identifies specialized NK cell subsets as the source of IFN-ɣ influencing the clinical efficacy of anti-HER2 antibodies. It also reveals the potential of serum CCL5/CXCL9 as biomarkers for identifying patients with NK cell-rich tumors and favorable responses to anti-HER2 antibody-based neoadjuvant treatment.

**Supplementary Information:**

The online version contains supplementary material available at 10.1186/s13046-023-02918-4.

## Background

Overexpression of the human epidermal growth factor receptor 2 (HER2) defines a subgroup of breast tumors with aggressive behavior [1]. Combination of chemotherapy and anti-HER2 monoclonal antibodies, trastuzumab plus pertuzumab is the prevailing neoadjuvant approach for patients with primary HER2-positive breast cancer. Achievement of pathological complete response (pCR) to neoadjuvant treatment has been associated with improved disease-free and overall survival. However, 35–50% of patients do not achieve pCR and/or eventually relapse [2]. One of the mechanisms contributing to the efficacy of anti-HER2 antibodies is the triggering of Natural Killer (NK) cell-mediated antibody-dependent cell cytotoxicity (ADCC) [3].

NK cells are cytotoxic innate lymphocytes that can directly eliminate cancer cells. When bound to HER2 expressing targets, anti-HER2 antibodies of the IgG1 subtype can trigger NK cell activation through the FcγRIIIa (CD16) leading to cytotoxic granule release towards target cells, and the secretion of IFNγ, TNFα and T cell-recruiting chemokines [4]. These mechanisms of action lead to tumor cell lysis and may contribute to the development of subsequent anti-tumoral adaptive immune responses [1, 5, 6]. We have recently evidenced that the number of tumor-infiltrating NK cells (TI-NK) in diagnostic biopsies was associated to the achievement of pCR and prolonged disease-free survival upon neoadjuvant treatment with anti-HER2 antibodies, independently of conventional clinicopathological factors [7]. Despite showing a remarkable potential as a predictor of response, the number of TI-NK cells in tumor biopsies was scarce, suggesting a putative role of TI-NK cells as regulators of the tumor microenvironment, in addition to their cytotoxic function.

NK cells belong to the innate lymphoid cell family (ILC) that also includes tissue-resident non-cytotoxic lymphocyte subsets with regulatory function. Yet NK cells appear to be the most abundant ILC, non-cytotoxic ILC1 as well as ILC3 cells have also been described in human and mouse breast tumors [8]. Whereas the association between NK cell infiltration and improved prognosis has been observed in many tumor types [9], the contribution of non-cytotoxic ILC to anti-tumor immunity remains controversial showing pro- and anti-tumorigenic roles in a context and model-dependent manner [8]. In addition, tumor-infiltrating NK cells undergo phenotypic and functional alterations owing to the influence of the tumor microenvironment, adding difficulty to the delineation of each specific ILC subset [10–13].

The cooperation between CCL5 and the inducible CXCL9 and CXCL10 chemokines guides the infiltration of CXCR3^+^ NK and T cells into solid tumors [14–16] and associates to favorable responses to chemotherapy and immunotherapy with immune checkpoint blockers [17–21], but how these mechanisms are orchestrated in response to anti-HER2 antibody-based treatment remains unaddressed.

## Methods

### Human samples and ethics statement

Gene expression microarray analysis were performed from 6 TI-NK cell positive and 6 TI-NK cell negative HER2-positive breast tumor biopsies, selected from a previously described cohort, based on double CD3 CD56 immunohistochemistry data [7].

Treatment-naïve, fresh, breast tumor specimens were, mechanically disrupted and digested for 40 min at 37ºC with 1% FBS, collagenase type IV (1 mg/ml, 171040-19, Gibco) and DNAse (50 ×10^3 ^U/ml, M0303, New England Biolabs). Tissue clogs were removed by filtering prior to immunostaining of the cell suspension with directly labeled specific antibodies as detailed below.

Peripheral blood mononuclear cells (PBMC) were obtained from volunteer healthy adults or breast cancer patients by Ficoll-Hypaque gradient (Lymphoprep, 10718463, Stemcell) and kept overnight in complete RPMI 1640 GlutaMAX (72400-021, Gibco) [i.e. penicillin/streptomycin (100 U/ml and 100 μg/ml, respectively, 15140–122 Gibco), sodium pyruvate (1 mM, 11360–070, Gibco), 10% FBS (26140-079, Gibco)] supplemented with recombinant IL-2 (200 U/ml, Proleukin). NK cells were purified by negative selection using NK isolation kit (130–092-657, Miltenyi) according to the manufacturer instructions.

Serum samples were obtained from HER2^+^ breast cancer patients undergoing neoadjuvant treatment. Serum was aliquoted and stored at -80ºC until analysis. Patients included newly diagnosed, previously untreated, primary breast cancer cases from Hospital del Mar, Barcelona and Hospital Clínic, Valencia. HER2-positive subtype classification was defined following 2013 ASCO/CAP guidelines [22]. All patients received a neoadjuvant combination therapy of standard chemotherapy and anti-HER2 mAbs. Pathological response at surgery after completing the neoadjuvant treatment was reported according to Miller-Payne grading system. Grades 1–4 are categorized as a partial pathological response and grade 5 (no malignant invasive cells identifiable in sections from the site of the tumor and axillar lymph nodes; only vascular fibroelastotic stroma) as pathological complete response (pCR).

### Gene expression microarray analysis of FFPE breast tumor biopsies

Gene expression microarray analysis between TI-NK cell-positive and -negative tumors was performed by the MARGenomics core facility at Hospital del Mar Medical Research Institute, Barcelona. Details on procedures and analysis are described in supplementary methods.

### Cell lines and in vitro ADCC assays with purified NK cells

Details on cell lines used in this study and in vitro ADCC assays are included in Supplemental methods.

### Treatment of breast cancer cells with recombinant cytokines

SKBR3 and HC1954 cells were treated with recombinant human rhIFN-ɣ (10 ng/ml, 300–02, Peprotech), rhTNF-α (10 ng/ml, 1134013, Immunotools), rhIFN-β-1a (1000 U/ml, Rebif, Merck) and their combinations for 24 h prior to supernatant collection and chemokine analysis by ELISA.

### CCL5, IFN-γ, CXCL9 and CXCL10 analysis by ELISA

CCL5, CXCL9 and CXCL10 levels were measured with DY278, DY392 and DY266, R&D commercial ELISA, respectively, following the manufacturer’s instructions. IFN-γ, levels were measured with 88–7316-88, Invitrogen, commercial ELISA, also following the manufacturer’s instructions. For serum samples, an interassay consistency test was performed by correlating the values obtained for each chemokine in the analysis of different aliquots from the same serum samples in two independent ELISA.

### TILs and TI-NK cell determination by IHC

TIL score and tumor-infiltrating NK cell numbers in breast tumor biopsies were obtained from a previously characterized cohort [7].

### A humanized mouse model to study in vivo NK cell-mediated ADCC

All animal experiments were performed in accordance with protocols approved by the Barcelona Biomedical Research Park (PRBB) Animal Facility and Generalitat de Catalunya Animal Care and Use Committee (EARA-20–0045). Eight weeks old NOD/Scid/γc-/- (NSG) mice were obtained from Jackson laboratories (005557). Tumors were induced by subcutaneous injection of human HCC1954 cells (4 × 10^5^) embedded in matrigel into the right flank. Tumor size was calculated using the formula (width^2^ × length x π)/6. When tumors reached 100 mm^3^ size, mice were treated with either: i) control hIgG1 (Rituximab; 2 mg/Kg) ii) trastuzumab and pertuzumab (1 mg/Kg, each), iii) expanded human NK cells (2.5 x 10^6^ cells) or iv) expanded human NK cells and the trastuzumab/pertuzumab combination (*n* = 5 mice/condition). All antibodies were administered intraperitoneally every 3–4 days. NK cells were expanded as described in supplemental methods and injected intratumorally once per week over three weeks, along with recombinant human IL-2 (rhIL-2, 200 IU/mice) and sustained by intraperitoneal rhIL-2 (20.000 IU/mice) every 3–4 days. After completing three treatment cycles, mice were sacrificed and the remaining tumors were collected and digested. Half of the obtained cell suspensions were used for RNA extraction and analysis of *CCL5, IFNG, CXCL9/10* expression whereas the other half were used for flow cytometry analysis as detailed in supplemental methods.

### Characterisation of breast tumor immune infiltrates by multiparametric flow cytometry

Multicellular suspensions obtained from fresh breast tumor digestion were pretreated with aggregated human IgG (10 μg/ml) and subsequently stained with a combination of directly labelled antibodies. Antibodies used included: Anti-CD45-AlexaFluor700 (clone 2D1, 56–9459-42), anti-CD16-APC-efluor780 (clone CB16, 47–0168-42), anti-CD103-FITC (clone B-Ly7, 11–1038-42) and anti-PD1-efluor610 (clone J105, 61–2799-42) from eBiosciences; anti-CD3-PerCP (clone SK7, 345766) and anti-CD8-HV500 (clone RPA-T8, 560775) from BD Biosciences; anti-CD4-PECy7) (clone OKT4, 317414) fom Biolegend); anti-NKG2C-PE (clone 134591, FAB138P) from R&D and anti-CD56-APC (clone CMSSB, 17–0567-42) from Invitrogen. Data was acquired on a BD LSR Fortessa or BD-LSRII (BD Bioscience) and analyzed with FlowJo software (v10.0.7, Tree Star). Details on vi-SNE analysis are described as supplemental methods.

### Ex vivo treatment with anti-HER2 antibodies of multicellular cultures derived from patient breast tumors 

Multicellular suspensions obtained from fresh breast tumor digestion were culture in flat bottom 96-well plates (153596, Corning) at a density of 100,000 cells/well for 20–24 h with 200 U/ml of rhIL2 and with or without 210 ng/ml of trastuzumab (Tz, from Hospital del Mar Pharmacy). After culture, cells were stained with a combination of directly labeled antibodies, which included: anti-CD45 (clone 2D1, 56–9459-42, eBiosciences), anti-CD56 (clone CMSSB, 17–0567-42, Invitrogen), anti-CD3 (clone SK7, 345766, BD), anti-CD16 (clone CB16, 47–0168-42, eBiosciences), anti-CD103 (clone B-Ly7, 11–1038-42, eBiosciences) and anti-CD137 (clone 4B4, 12–1379, eBiosciences). Data was acquired on a BD LSR Fortessa (BD Bioscience) and analysed with FlowJo software.

### Sorting of circulating and tumor-infiltrating NK cell subsets and bulk RNAseq analysis

Paired samples of peripheral blood and treatment-naive breast tumor specimens were obtained from three breast cancer patients, blind to their clinicopathological features. Multicellular suspensions from tumors were obtained as described above and stained with a directly labelled antibody cocktail including: a-CD45-AF700 (clone 2D1, 56–9459-42, eBiosciences), a-CD3-PerCP (clone SK7, 345766, BD), a-CD56-APC (clone CMSSB, 17–0567-42, Invitrogen), a-CD16-efluor780 (clone CB16, 47–0168-42, eBiosciences) and a-CD103-FITC (Clone B-Ly7, 11–1038-42, eBiosciences). DAPI was used as a viability die. Distinct tumor-infiltrating NK cell subsets were sorted based on the expression of CD16 and CD103 (CD16^+^; CD16^-^CD10 ^+^; CD16^-^CD103^-^) from the CD56^+^ CD3 gate in CD45^+^ DAPI^-^ lymphocytes. Peripheral blood NK cells were sorted from PBMC based on their CD56^bright^ CD16^-^ and CD56^dim^ CD16^+^ expression profile. Cell sorting was performed in a FACS Aria II SORP cell sorter (23–9641-01, BD) at the Flow Cytometry Facility, PRBB, Barcelona. RNA-Seq sample processing and analysis were performed by Pompeu Fabra University Genomic Core Facility, Barcelona. GSEA analysis was performed by Mar Genomics Facility at IMIM, Barcelona. Procedures and analysis are included as supplementary methods.

### Bioinformatic analysis of publicly available gene expression data from HER2^+^ breast tumors

Gene expression microarray data from treatment-naive HER2-positive breast tumors obtained from breast cancer patients undergoing trastuzumab-based neoadjuvant treatment in a Phase II randomized clinical trial was downloaded from the Gene Expression Omnibus (GEO, https://www.ncbi.nlm.nih.gov/geo/; GSE130786 dataset). Agilent array data was quantile normalized and log2 transformed. All data was processed within R. Single sample score of NK signature (*CD160, CD244, CTSW, FASLG, GZMA, GZMB, GZMH, IL18RAP, IL2RB, KIR2DL4, KLRB1, KLRC3, KLRD1, KLRF1, KLRK1, NCR1, NKG7, PRF1, XCL1 and XCL2* genes) [23] was computed with the singscore R package. Z scores for *GZMB, IFNG, CCL5, CXCL9, CXCL10* transcripts were used for analysing their association pCR to trastuzumab treatment. Odds ratio for pCR achievement were calculated for the top and bottom quartile expression ranked values of the selected genes or the ranked sum expression of the NK gene signature.

## Results

### The *CCL5/IFNG-CXCL9/CXCL10* axis links TI-NK cells to the effectiveness of neoadjuvant anti-HER2 antibody-based treatment

**Fig. 1:**
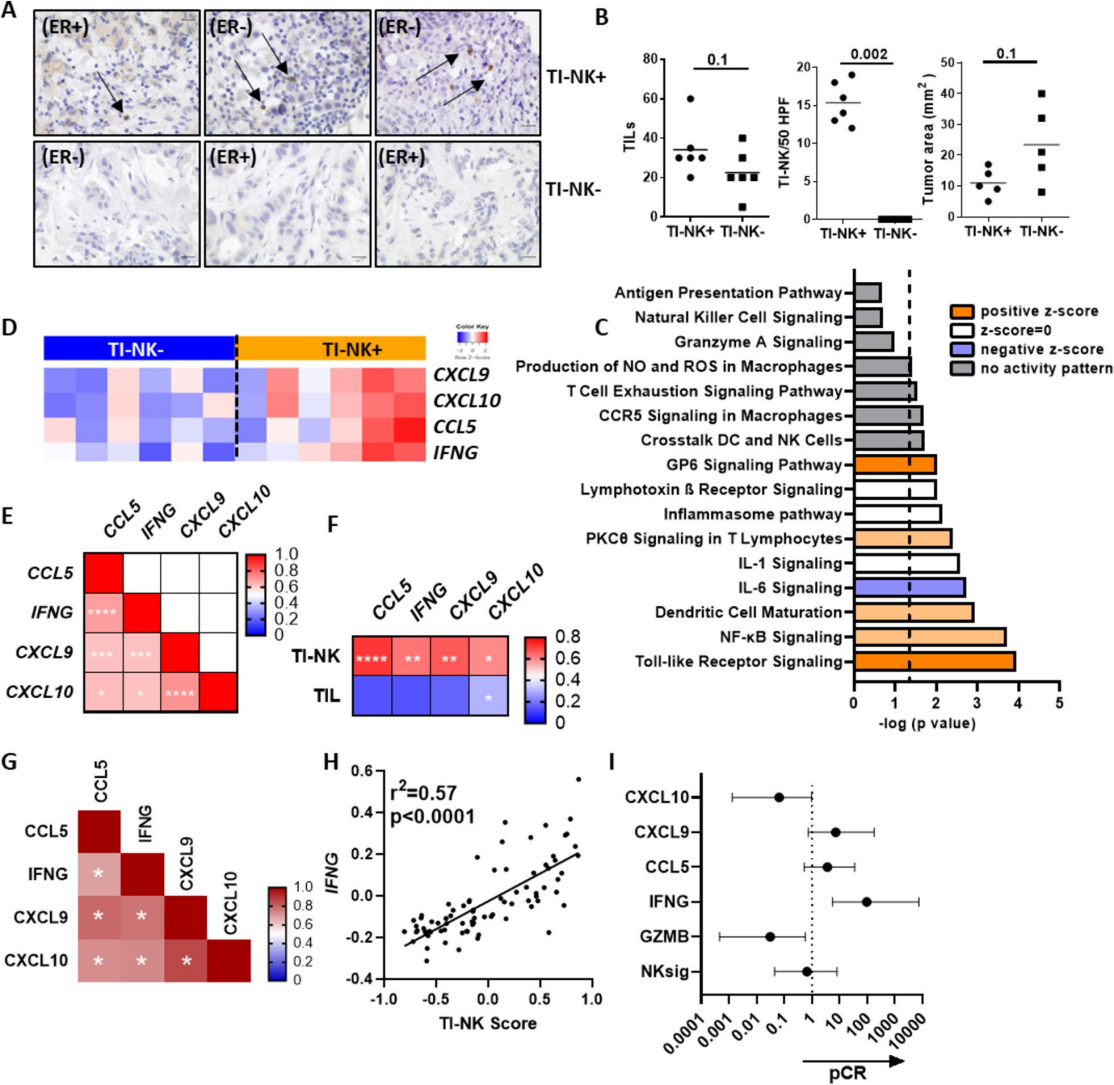
Association of *CCL5/IFNG-CXCL9/10* axis, TI-NK cell signature and response to neoadjuvant treatment with anti-HER2 antibodies. **A** Images showing CD56 staining by IHC of representative tumors included in the array. Arrows point to CD56 + lymphocytes. ER: Estrogen Receptor. **B** TIL score, TI-NK cell number and tumor area in the analysed tumor biopsies (*n* = 12). **C** Biological pathways enriched in TI-NK cell infiltrated tumors. Dashed line corresponds to -log (*p* value) = 1.3. **D** Relative expression of *CCL5*, *IFNG*, *CXCL9* and *CXCL10* genes in all tumors included in the array. **E** Pearsons’ correlation coefficient between indicated genes. **F** Pearsons’ correlation coefficient between indicated genes, TI-NK cell numbers and TIL scores in the corresponding biopsies. Asterisks indicate statistical significance (**** *p* < 0.0001; *** *p* < 0.001; ** *p* < 0.01; * *p* < 0.05). **G-I** A gene signature predicting NK cell infiltration (see methods), *IFNG, GZMB, CCL5, CXCL9* and *CXCL10* gene expression levels were analysed in treatment naïve HER2-positive breast tumors from the Phase II Trial dataset (GSE130786). **G** Pearson correlation coefficient between indicated genes. Asterisk label significant correlations. **H** Correlation between *IFNG* expression levels and TI-NK cell score. **I** Odds Ratio (OR) and 97.5 confidence interval (CI) of TI-NK cell (NKsig), *IFNG, GZMB, CCL5*, *CXCL9* and *CXCL10* scores for pCR to trastuzumab. Asterisk label significant genes

The presence/absence of tumor-infiltrating NK cells (TI-NK cells) in treatment-naive breast tumor biopsies was associated to benefit/resistance to anti-HER2 antibody-based neoadjuvant treatment in HER2^+^ breast cancer patients with primary disease [7]. To obtain a global view of the biological processes associated to NK cell infiltration, we compared the transcriptomes of NK cell-enriched (*n* = 6) as compared to NK cell-desert (*n* = 6) diagnostic biopsies of HER2^+^ tumors by microarray analysis. The included biopsies were discordant for TI-NK cell content and response to treatment with anti-HER2 antibodies, but matched by patient age, tumor area and TIL score (Fig. 1A, B and Supplementary Fig. 1A). There were 130 differentially expressed genes (DEG) in TI-NK cell-rich as compared to TI-NK cell-desert tumors, including 42 down-regulated and 88 up-regulated genes (Supplementary Fig. 1B). Canonical Pathway Analysis of DEGs identified an enrichment in GP6, PKCθ, TLR, NF-ƙB signaling and dendritic cell maturation biological processes in NK cell-infiltrated breast tumors, which also showed negative scores for IL-6 signaling-related genes (Fig. 1C). Analysis of gene regulatory networks pointed to IFN-γ, TNF and type I IFNs as major regulators of DEG, which converged in the coordinated upregulation of CXCL9 and CXCL10 in TI-NK cell-rich tumors (Supplementary Fig. 1C and Fig. 1D). Indeed, the expression of *CCL5, IFNG, CXCL9* and *CXCL10* positively correlated among themselves as well as with the numbers of tumor-infiltrating NK cells in the original biopsy (Fig. 1E–F). Analysis of these 4 genes in a public gene expression dataset from HER2-positive breast tumors generated in the context of a Phase II Randomized Trial (GSE130786, *n* = 81) corroborated our original findings. Also, in this larger dataset, transcript levels for *CCL5, IFNG, CXCL9* and *CXCL10* were positively correlated to each other in baseline biopsies (Fig. 1G). In multivariate logistic regression analysis, *IFNG* relative expression was positively correlated with TI-NK cell gene signature (Fig. 1H) and the probability of achieving pCR to trastuzumab-based neoadjuvant treatment (OR 96.3, *p* = 0.01) (Fig. 1I). These results point to NK cells as the original source of IFN-ɣ and indicate the importance of *CCL5/IFNG-CXCL9/10* axis for the clinical efficacy of anti-HER2 antibody-based treatment in primary HER2 + breast cancer patients.

### IFN-ɣ produced along anti-HER2 antibody-dependent NK cell activation triggers the production of CXCL9/10 by breast cancer cells

**Fig. 2:**
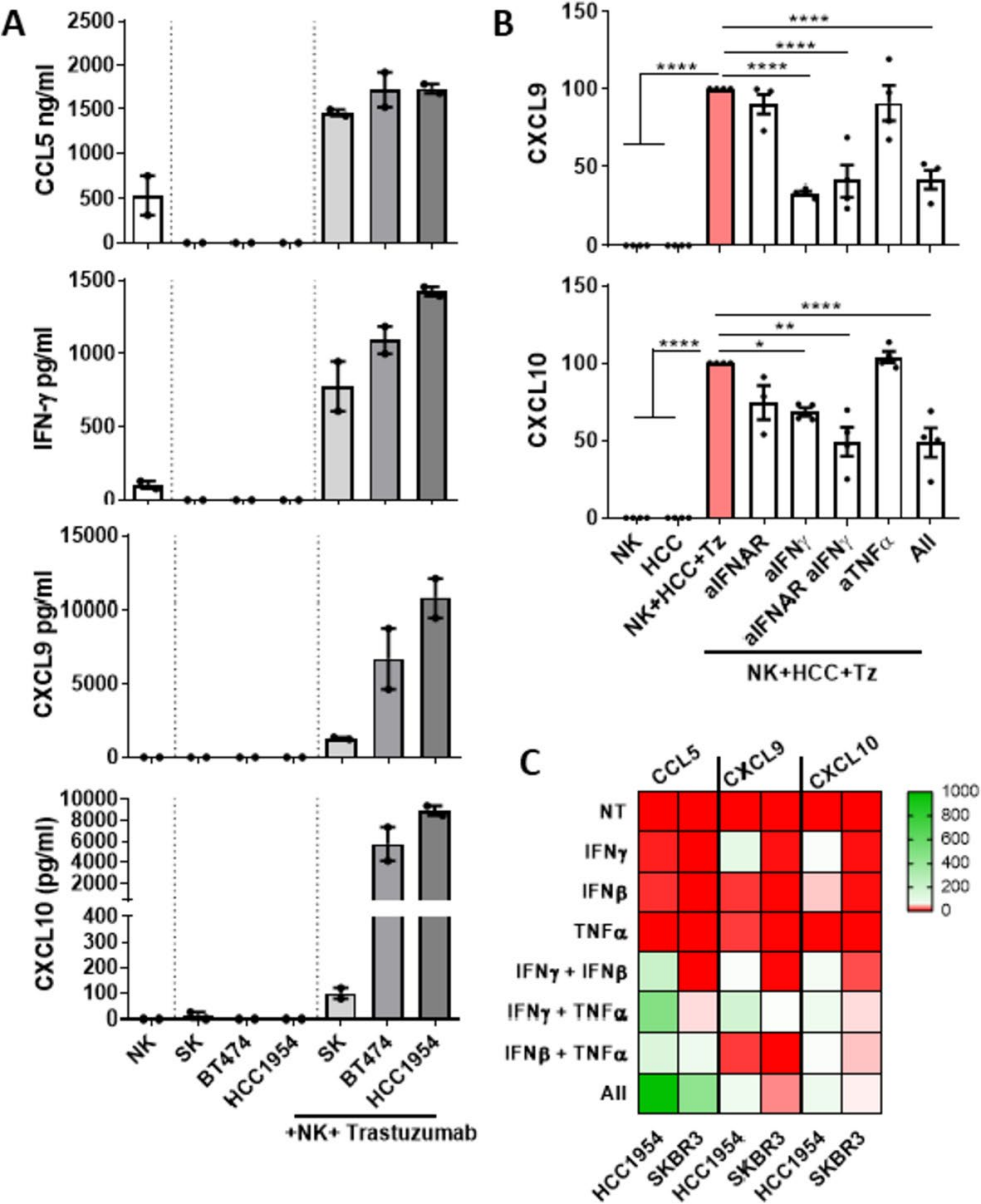
Anti-HER2 antibody-dependent NK cell activation triggers the CCL5/IFN-CXCL9/10 axis in in vitro ADCC assays. **A**, **B** Purified primary NK cells were cocultured with SKBR3, BT474 and HCC1954 breast cancer cell lines (E:T ratio 1:1) in the presence of trastuzumab (210 ng/ml) for 24 h. In some experiments blocking/neutralizing antibodies for IFNAR (5 µg/ml), IFN-ɣ (5 µg/ml) and TNF-α (50 µg/ml) were included in the culture. CCL5, IFN-ɣ, CXCL9 and CXCL10 production was analysed in cell-free culture supernatants by ELISA. **A** Average amount of CCL5, IFN-ɣ, CXCL9 and CXCL10 in each indicated condition, in two independent experiments. **B** Production of CXCL9 and CXCL10 upon IFN-ɣ, TNF-α and IFNAR individual or combined blockade in in vitro ADCC assays, as analysed by ELISA. Data from three independent experiments normalized to the production of each cytokine/chemokine in the absence of blocking antibodies. Asterisks label statistically significant differences by Mann Withney U test. **C** HCC1954 and SKBR3 cells were treated with recombinant IFN-ɣ (10 ng/ml), TNF-α (10 ng/ml), IFN-β (1000 U/ml) and their combinations for 24 h. Cell culture supernatants were analysed for the presence of CCL5, CXCL9 and CXCL10 by ELISA. HeatMap showing the absolute amount each chemokine in each condition in pg/ml for CCL5 and ng/ml for CXCL9 and CXCL10

We next addressed whether cytokines produced during trastuzumab-dependent NK cell-activation could stimulate CXCL9/10 production from bystander breast cancer cells in in vitro coculture assays. Trastuzumab-induced NK cell activation against SKBR3, BT474 and HC1954 HER2-positive breast cancer cells resulted in the secretion of CCL5, IFN-γ and the consequent production of CXCL9 and CXCL10, as analysed by ELISA in cell-free coculture supernatants (Fig. 2A). Assays including a blocking antibody for type I IFNs (anti-IFNAR) or neutralizing antibodies for IFN-γ and TNF-α showed that CXCL9 secretion by HCC1954 cells was mostly driven by IFN-γ while both type I and type II IFNs contributed to CXCL10 production (Fig. 2B). Comparable results were obtained with the SKBR3 cell line (Supplementary Fig. 2). Certainly, in vitro treatment of HCC1954 and SKBR3 cells with recombinant cytokines confirmed a major role for IFN-γ in regulating CXCL9 and CXCL10 secretion. Additionally, it revealed a cooperative effect between IFN-γ and TNF-α in inducing the production of CCL5 and CXCL9 from breast cancer cells (Fig. 2C). Of note, the effect of IFN-β was chemokine-dependent, showing a modest capacity for inducing CXCL10, a cooperative effect with IFN-γ and TNF-α for inducing CCL5 secretion while counteracting the effect of IFN-γ and TNF-α on CXCL9 secretion (Fig. 2C). As detected along trastuzumab-dependent NK cell activation in in vitro assays, there was a high variability in the levels of chemokines released in response to IFN-γ by the different breast cancer cell lines (Fig. 2A and C). These results indicate that the interaction of NK cells with breast cancer cells along anti-HER2 antibody-dependent ADCC is enough for unleashing the secretion of CCL5/IFN-ɣ-CXCL9/10 axis.

### The activation of CD16^+^ NK cells by anti-HER2 antibodies associates with tumor growth control, IFN-ɣ production and the acquisition of CD103 expression

**Fig. 3:**
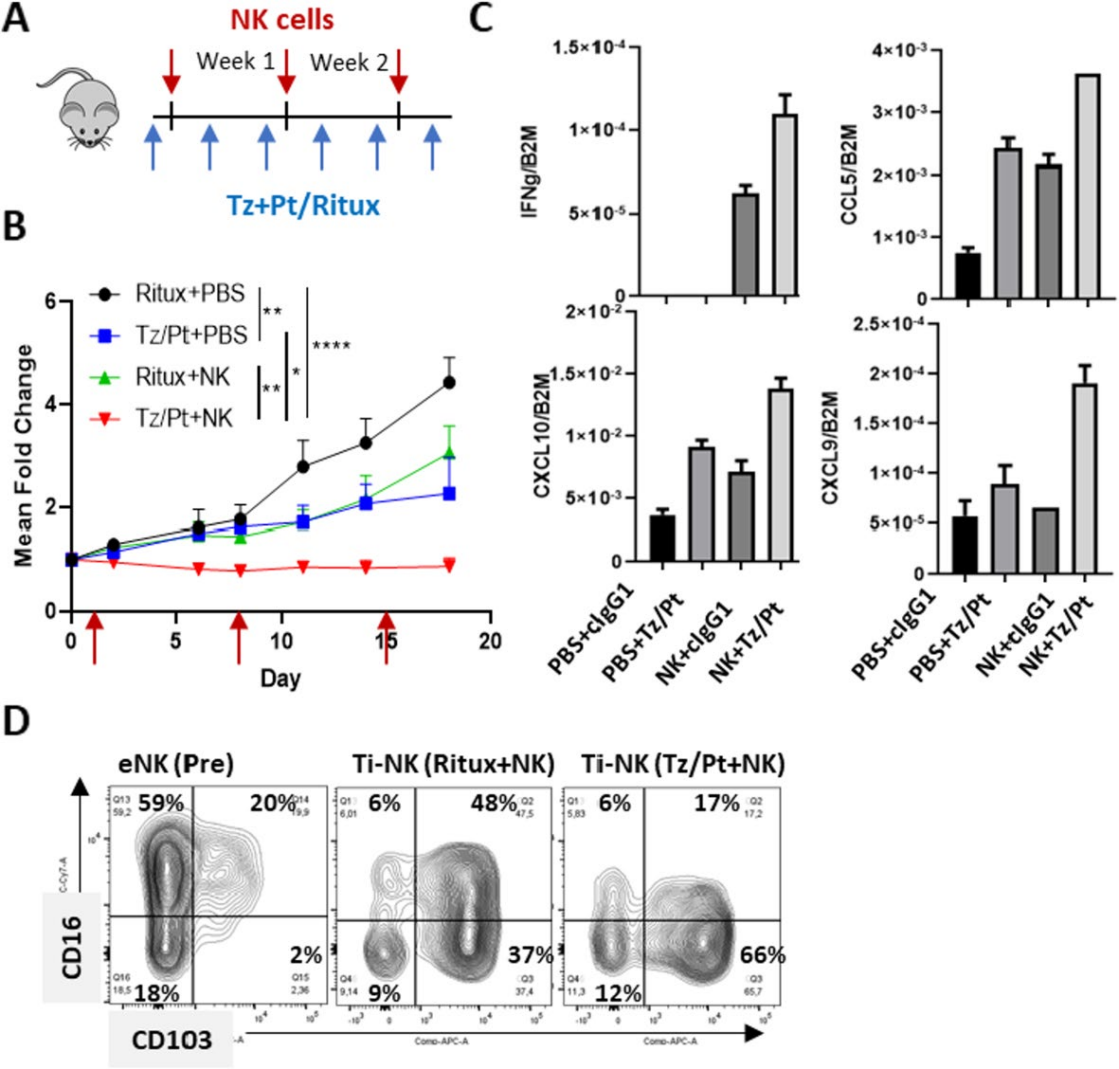
NK cell and anti-HER2 antibody combined treatment in a humanized mice model of HER2-positive breast cancer. HCC1954 cells (4 × 10^5^) were subcutaneously implanted in NSG mice. When tumors reached 100 mm^3^ mice were treated with either: i) isotype control (rituximab; 2 mg/Kg intraperitoneal); ii) trastuzumab (Tz)/pertuzumab (Pt) (1 mg/Kg each, intraperitoneal); iii) expanded human NK cells (2.5 × 10^6^, intratumoral); or iv) trastuzumab/pertuzumab in combination with expanded human NK cells. Mice were sacrificed at the end of treatment. Remnant tumors were excised and processed for RNA extraction and immunostaining of tumor-infiltrating NK cells as indicated in methods. **A** Treatment schedule. **B** Tumor volume fold change in each treatment group (*n* = 5 mice/group). Statistical significance by two-way ANOVA test. **C** *CCL5, IFNG, CXCL9* and *CXCL10* expression by RT-qPCR in total tumor RNA extracts in each treatment group. **D** CD16 and CD103 surface expression in expanded NK cells prior to intratumoral injection (eNK; pre) as well as in tumor-infiltrating NK cells (TI-NK) for the indicated treatment groups

To address whether systemic treatment with anti-HER2 antibodies could indeed result in the activation of TI-NK cells and the triggering of CCL5/IFN-γ -CXCL9/10 axis, we established a humanized in vivo model. HCC1954 xenografts were subcutaneously implanted in NOD/Scid/γc-/- (NSG) mice and treated with either: i) intraperitoneal injections of anti-HER2 antibodies (trastuzumab and pertuzumab), ii) intraperitoneal injections of control hIgG1 (rituximab), iii) intratumoral injections of expanded human NK cells in combination with intraperitoneal control antibody or iv) combination of intraperitoneal anti-HER2 antibodies and intratumoral expanded human NK cells (Fig. 3A). Compared to control groups, mice receiving the combined treatment with human NK cells and anti-HER2 antibodies showed complete tumor growth inhibition, surpassing the efficacy of trastuzumab/pertuzumab dual treatment or of NK cell monotherapy (Fig. 3B). At the end of treatment, remaining tumors were excised and total RNA obtained. *IFNG* transcripts were exclusively detected in tumors that received NK cell treatment (Fig. 3C). Tumors from animals treated with systemic anti-HER2 antibodies or intratumoral NK cells as monotherapy showed increased expression of *CCL5* and *CXCL10* but not of *CXCL9* transcripts, compared to non-treated tumors. A coordinated increase in *CCL5/IFNG-CXCL9/10* levels was only detected in tumors treated with the combination of NK cells and anti-HER2 antibodies, supporting the requirement of both elements for effectively triggering the CCL5/IFN-γ -CXCL9/10 axis (Fig. 3C). Phenotypic characterization of tumor-infiltrating NK cells from resected tumors showed that, in comparison to the pre-injected NK cell product (CD16^+^CD103^-^ NK cells), intratumoral NK cells displayed a progressive acquisition of CD103 (CD16^+^CD103^+^) concomitant with CD16 loss (CD16^-^CD103^+^ NK cells) (Fig. 3D). This effect was proportional to the magnitude of the activating stimuli and the control of tumor growth (Fig. 3D, B). Overall, these results showed that CD16^+^ NK cells play a direct role in suppressing tumor progression in the presence of anti-HER2 antibodies. Remarkably, this effect was associated to the triggering of *CCL5/IFNG-CXCL9/10* axis and with changes in their phenotype reminiscent to intra-epithelial ILC1.

### Specialized tumor-infiltrating NK cell subsets associate to distinct immune contextures in human breast tumors

**Fig. 4:**
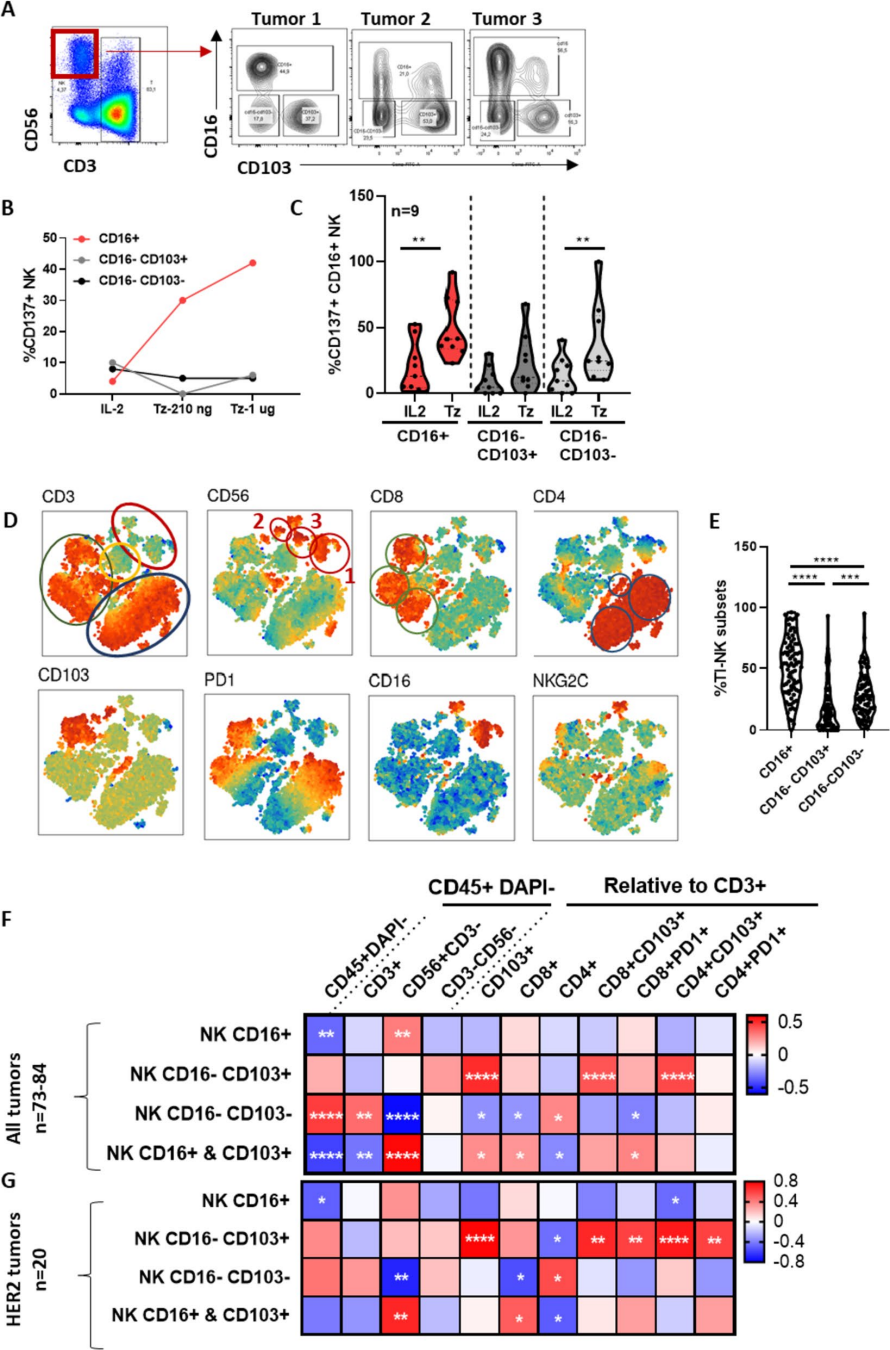
Specialized tumor-infiltrating NK cell subsets associate to distinct immune contextures in human breast tumors. Unsorted multicellular suspensions derived from fresh breast tumor specimens were cultured in complete medium with IL2 in the presence or absence of trastuzumab (Tz; 210 ng/ml) for 24 h. **A** Dot plots showing the NK cell gate (CD56^+^CD3^-^) used for the analysis of the expression of CD16 and CD103 in TI-NK cells of three representative samples. **B-C** Proportions of CD137^+^ cells in CD16^+^, CD16^-^CD103^+^ and CD16^-^CD103^-^ subpopulations in fresh breast tumor-derived multicellular cultures treated or not with trastuzumab. **D-G** Fresh breast tumor specimens (*n* = 84) were processed and stained with a combination of antibodies specific for CD45, CD3, CD56, CD8, CD4, CD16, CD103, NKG2C and PD1 and analysed by flow cytometry. **D** tSNE of major alive lymphocyte subsets (DAPI^-^CD45^+^) in breast tumor immune infiltrates generated with data from 18 tumors. Red, blue and green circles indicate major NK, CD4 and CD8 T cell subsets, respectively. **E** Percentage of CD16^+^ , CD16^-^ CD103^+^ and CD16^-^CD103^-^ TI-NK cell subpopulations in treatment naïve, fresh tumor samples. **F**, **G** Heat maps showing Spearman’s correlation coefficients between the indicated lymphocyte subsets in breast tumors (*n* = 73–84) and HER2 tumors (*n* = 20). Asterisks label significant correlations (**** *p* < 0.0001; *** *p* < 0.001; ** *p* < 0.01; * *p* < 0.05

Based on observations from our in vivo model, we analysed the expression of CD16 and CD103 in tumor-infiltrating NK cells from fresh, treatment-naïve, human breast tumor specimens by flow cytometry. Differential expression of CD16 and CD103 discriminated three subpopulations within the conventional NK cell gate (CD56^+^ CD3^-^ lymphocytes): CD16^+^ cells with variable expression of CD103, CD16^-^CD103^+^ and CD16^-^CD103^-^ subsets (Fig. 4A). In vitro treatment of human breast tumor-derived multicellular cultures with anti-HER2 antibodies induced the activation of CD16^+^ NK cells in a dose-dependent manner, as evidenced by the up-regulation of CD137 (Fig. 4B, C). In some cultures, upregulation of CD137 upon trastuzumab treatment was also detected in CD16^-^ CD103^+^ and CD16^-^CD103^-^ TI-NK cell subsets (Fig. 4C).

We then sought to characterize the relationship between different TI-NK cell subpopulations and major T cell subsets in a cohort of prospectively recruited fresh breast tumor specimens (*n* = 84; cohort description in Supplementary Table 1). TIL content was analysed by multiparametric flow cytometry using a panel of antibodies to discriminate viable leukocytes (CD45+ DAPI-), CD3 and CD56 for T/NK cell gating, CD4 and CD8, CD103 and PD1 for the analysis of tissue-resident and exhausted lymphocytes, respectively, in addition to the NK cell activating receptors CD16 and NKG2C. An initial analysis included an unbiased clustering of alive TIL by multidimensional v-SNE, utilizing data from 18 randomly selected tumors (5 HER2 positive, 11 Luminal and 2 TNBC). Phenograph clustering and manual grouping identified 10 major lymphocyte subsets (Supplementary Fig. 3A, B). In addition to the three NK cell subsets already defined in the conventional NK cell gate (CD56+ CD3- lymphocytes), concomitant immune infiltrates included CD8 and CD4 T cell clusters further subdivided into CD103^+^PD1^+^, CD103^-^PD1^+^ and CD103^-^PD1^-^ cells, respectively. A fraction of lymphocytes negative for all markers was also found and interpreted as B cell cluster (Fig. 4D, Supplementary Fig. 3A, B). Data from these NK and T cell subsets were collected from all tumors and further analysed for their correlation. Among TI-NK cells, the CD16^+^ subset was predominant although variable proportions of CD103^+^CD16^-^ and CD16^-^ CD103^-^ cells were also detected in most samples (Fig. 4E). Proportions of CD16^+^ as well as the combination of CD16^+^ and CD16^-^CD103^+^ subsets positively correlated with the overall proportions of TI-NK cells in breast tumor infiltrates (Fig. 4F), indirectly supporting the association between the presence of CD16^+^ and CD16^-^CD103^+^ NK cells with the efficacy of anti-HER2 antibody-based neoadjuvant treatment. Of interest, CD16^+^ and CD103^+^ TI-NK cells negatively correlated with total CD4^+^ T cells while showing a positive correlation with total CD8 and tissue-resident CD103^+^ CD8^+^ T cell frequencies. Both lymphocyte subsets associated with improved prognosis in breast cancer. The strongest positive correlations were found between CD16^-^ CD103^+^ TI-NK cells and CD3^+^ CD103^+^ T cells including both CD8^+^ CD103^+^ and CD4^+^ CD103^+^ T cells (Fig. 4F). These findings, suggest that specific tumor microenvironments simultaneously imprint a tissue-residency program in both innate and adaptive lymphocytes. On the other hand, CD16-CD103- TI-NK cells were negatively correlated with total TI-NK cells and were associated with immune infiltrates characterized by decreased CD8^+^ and tissue-resident CD103^+^ T cell proportions, as well as higher CD4 T cell frequencies (Fig. 4F), supporting their association with a more regulatory immune contexture. The ad hoc analysis of the HER2 positive tumor cohort (*n* = 20) revealed comparable relationships among lymphocyte subpopulations (Fig. 4G). Indeed, the distribution of lymphocyte subsets was comparable across all breast tumor molecular subtypes (Supplementary Fig. 4).

In summary, CD16^+^ and CD16^-^CD103^+^ TI-NK cell subsets, reminiscent to TI-NK cells controlling tumor growth in in vivo models, associated with immune infiltrates enriched in tissue-resident memory T cells. In contrast, CD16-CD103- TI-NK cell subset, likely containing ILC3 cells, associated to immune infiltrates with high CD4/CD8 T cell ratios.

### In human breast tumors, CD16 and CD103 identify lineage-related NK cell subpopulations capable of producing CCL5 and IFN-ɣ

**Fig. 5:**
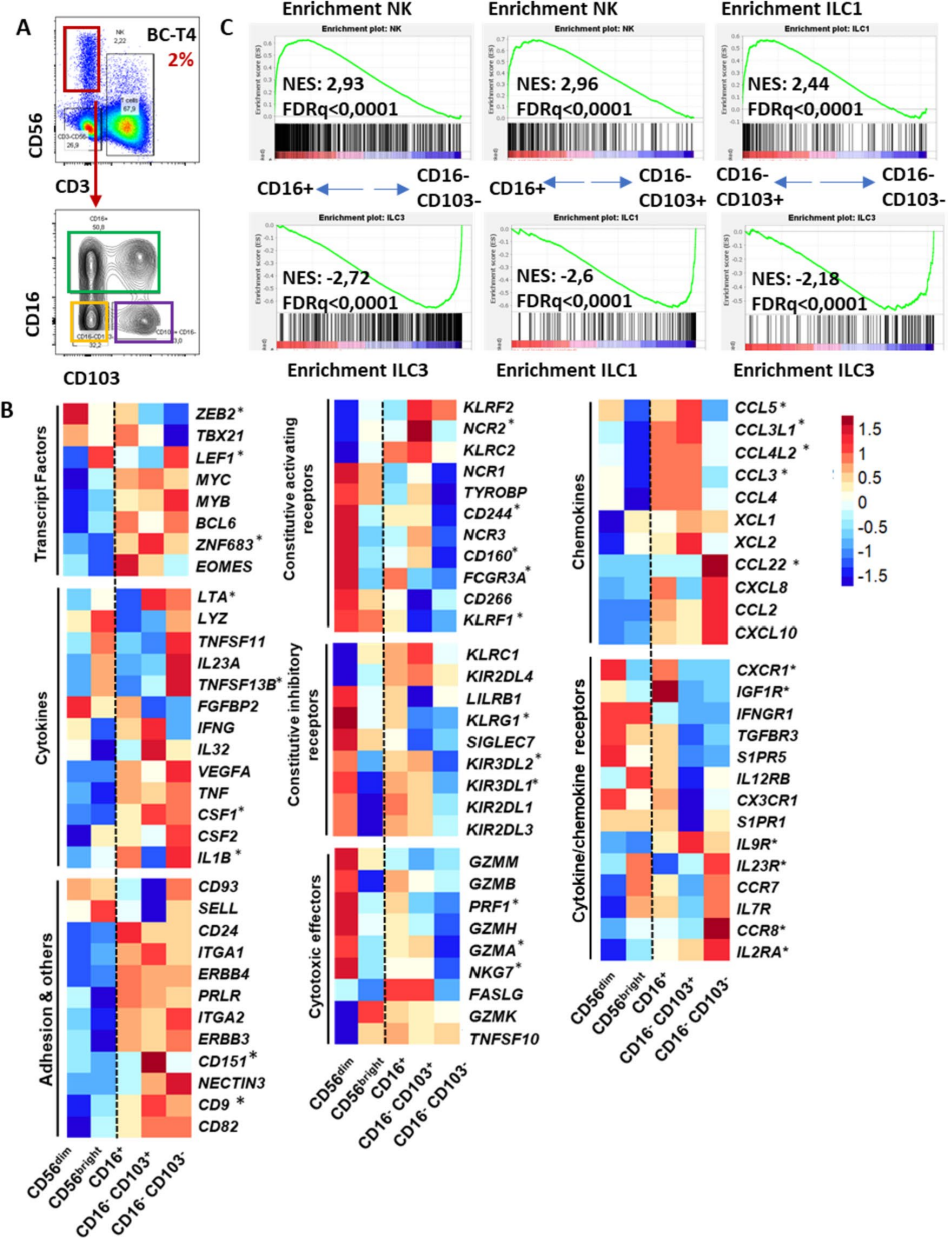
Transcriptomic characterization of sorted CD16^+^ , CD16^-^ CD103^+^ , CD16^-^ CD103^-^ TI-NK cell subsets in treatment naïve breast tumors. Total RNA was extracted from sorted CD16^+^, CD16^-^CD103^+^ and CD16^-^CD103^-^ TI-NK cells (CD56^+^ CD3- in DAPI^-^ CD45^+^ lymphocytes) from breast tumors as well as from CD56^bright^ CD16^-^ and CD56^dim^ CD16^+^ circulating NK cells from paired blood samples and analysed by RNAseq. Data from 3 patients. **A** Dot and density plot showing sorted TI-NK cell subsets in one representative tumor out of 3 analysed. **B** Log2 CPM mean expression (z-scores) of DEG in CD56^dim^ and CD56^bright^ peripheral blood NK cells, and CD16^+^, CD16^-^CD103^+^ and CD16^-^CD103^-^ TI-NK cells. Asterisk label differentially expressed genes in at least one of the three tumor-infiltrating NK cell subsets. **C** Gene set enrichment analysis (GSEA) of RNAseq data from TI-NK cell subsets against liver and intestine CD56^dim^ NK/ ILC1/ILC3: GSE37448; NES: normalized enrichment score. FDR: false discovery rate

We next analysed the functional profile of CD16^+^, CD16^-^CD103^+^ and CD16^-^CD103^-^ NK cells from three breast tumor samples upon sorting and subset-bulk RNA-seq. For comparison, CD56^dim^ and CD56^bright^ circulating NK cells were sorted based on CD56 and CD16 expression from paired peripheral blood samples (gating strategy in Fig. 5A and Supplementary Fig. 5A). The three TI-NK cell subsets showed remarkably similar transcriptomes (Supplementary Fig. 5B). Genes deregulated in a specific cell population ranged from 23 transcripts in CD16^+^, 51 in CD16^-^CD103^-^ and 68 in CD16^-^CD103^+^ TI-NK cells (Supplementary Fig. 5C).

Upregulated transcripts in CD16^+^ TI-NK cells corresponded to several essential NK cell molecules, including cell-lineage transcription factors (i.e. *ZEB2*, *EOMES* and TBET (*TBX21*)), cytotoxic effectors (*GZMB, PRF1, FASL, TNFSF10*), KIR receptors (*KIR2DL4, KIR3DL2, KIR3DL1, KIR2DL3*) and chemokine/cytokine receptors (i.e. *CXCR1*) (Fig. 5B). CD16^-^CD103^+^ TI-NK cells shared with CD16^+^ TI-NK cells the expression of transcripts for *EOMES* and inhibitory KIR yet showing a tissue-resident profile featured by the highest expression of the transcription factor HOBIT (*ZNF683*) and tissue/tumor retention molecules such as CD9 and CD151 (in addition to CD103), reminiscent of TGFβ-induced ILC1 and akin to tissue-resident memory T cells [24] (Fig. 5B). Remarkably, unique features of CD16^-^ CD103^+^ TI-NK cells included an enrichment in transcripts encoding for chemokines involved in immune/dendritic cell recruitment (*CCL5*, *XCL1* and *XCL2*) [5] in addition to the highest levels of *IFNG* transcripts (Fig. 5B). Lastly, CD16^-^CD103^-^ TI-NK cells showed high expression of *IL7R, IL23R, IL2RA, CCR7* and *CCR8* along with high expression of *LEF1*, *CSF2*, *CCL22* and *TNFSF13B* transcripts, pointing to the inclusion of ILC3 cells (Fig. 5B). Indeed, GSEA analysis of the distinct TI-NK cell transcriptomes against previously described profiles of circulating and tonsillar NK, ILC1 and ILC3 cells [25], further corroborated the resemblance between CD16^+^ TI-NK cells and circulating CD56^dim^ NK cells, CD16^-^ CD103^+^ TI-NK cells and tonsillar ILC1, while CD16^-^ CD103^-^ TI-NK cell subset was similar to immunoregulatory ILC3 (Fig. 5C).

Altogether, these data supported the lineage relationship between tumor-infiltrating CD16^+^ and CD16^-^ CD103^+^ NK cells in human breast tumors, both subsets capable of CCL5/IFN-ɣ production. In addition, it also evidenced the regulatory profile of CD16^-^ CD103^-^ subset, reminiscent to regulatory ILC3.

### An early increase in serum CCL5/CXCL9 identifies patients with NK cell-rich tumors and favorable responses to anti-HER2 antibody-based neoadjuvant treatment

**Fig. 6:**
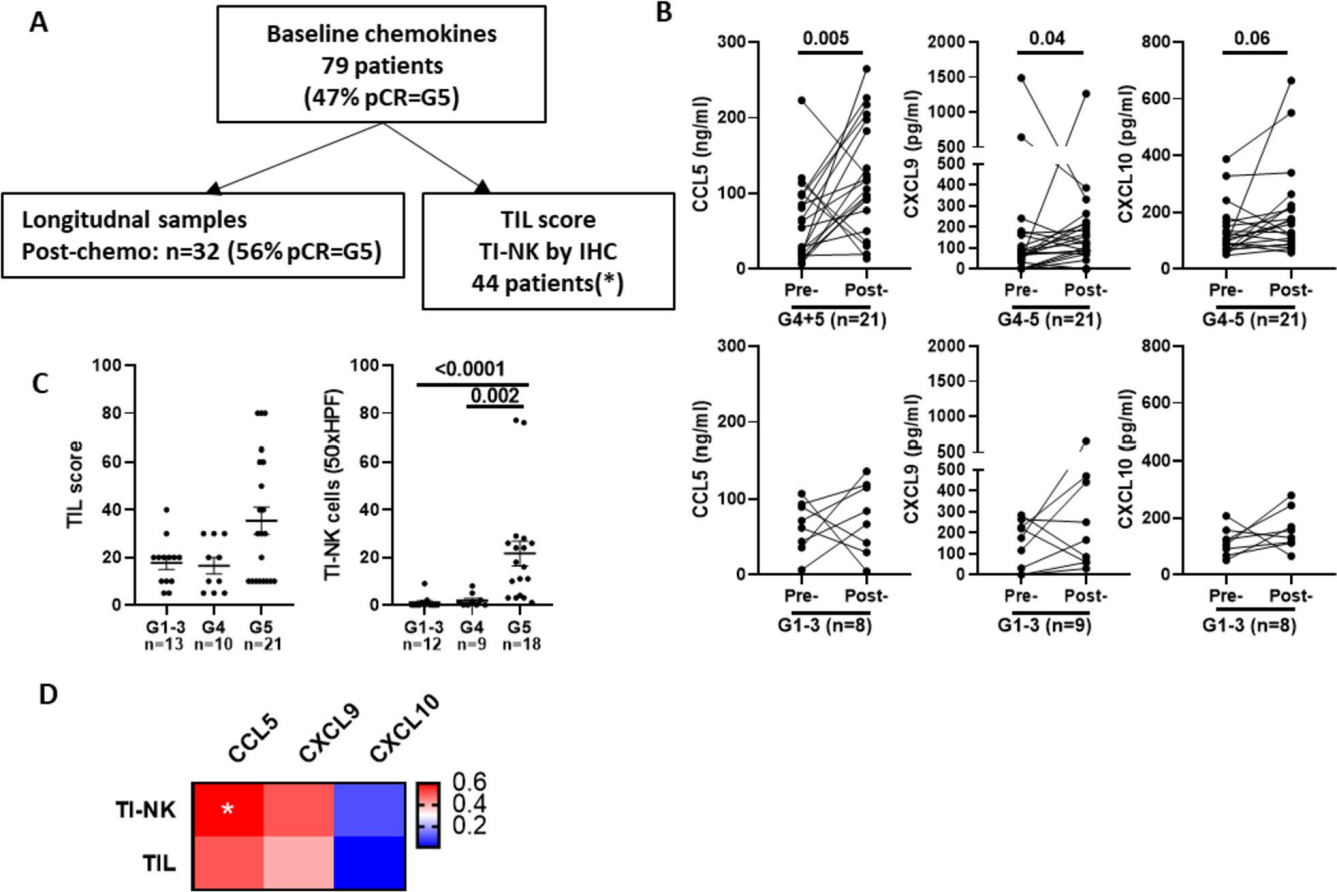
Changes in serum chemokine levels as early biomarkers of response to anti-HER2 antibody-based neoadjuvant treatment in HER2-positive primary breast cancer. CCL5, CXCL9 and CXCL10 levels were analysed by ELISA in longitudinal serum samples from HER2-positive breast cancer patients undergoing anti-HER2 antibody-based neoadjuvant treatment. Samples were obtained prior to treatment initiation (baseline, *n* = 79), after 3 chemotherapy cycles (Post-chemo; *n* = 32). **A** Scheme indicating the number of baseline and paired longitudinal serum samples for each time point; the percentage of pathological complete response (pCR) in each patient group; and patients with TILs and TI-NK cell data available. **B** CCL5, CXCL9 and CXCL10 levels in paired baseline (pre) and post-chemotherapy (post) serum samples in patients stratified as good (G4 and G5) or poor (G1-3) responders to anti-HER2 antibody-based neoadjuvant treatment by Miller and Payne criteria. **C** TIL score and TI-NK cell numbers in diagnostic biopsies of the analysed cohort, stratified by response to treatment according to Miller and Payne. Statistical significance by Mann Whitney U test is indicated. **D** Spearman correlation coefficient between baseline TIL score and TI-NK cell numbers and post-chemotherapy CCL5; CXCL9 and CXCL10. Significant correlations are indicated

We next addressed whether cytokines and chemokines related to TI-NK cell activation could be detected at systemic level. Prospective longitudinal serum samples were collected from HER2-positive breast cancer patients with primary disease who received HER2-antibody-based neoadjuvant treatment. Samples were collected at baseline (*n* = 79) and after three chemotherapy cycles (*n* = 32) (cohort description in Supplementary Table 2). Baseline TIL score and TI-NK cell numbers in the corresponding diagnostic biopsies were available for 44 of the included patients [7] (Fig. 6A). Initial tests showed that IFN-γ was not detected whereas the detection of CCL5, CXCL9 and CXCL10 in serum samples was highly reproducible in independent ELISA tests (Supplementary Fig. 6A). In contrast to tumor gene expression, the levels of systemic CCL5, CXCL9 and CXCL10 in baseline serum samples were similar among all patients, regardless of their response to anti-HER2 antibody-based neoadjuvant treatment (Supplementary Fig. 6B). However, a coordinated increase in systemic CCL5 and CXCL9 levels was detected after three chemotherapy cycles in patients who exhibited a good response to treatment (G5 and G4) (Fig. 6B). For those patients with available data, TI-NK cell numbers in baseline biopsies were higher in patients achieving pCR (G5) (Fig. 6C) and showed a positive correlation with systemic CCL5 and CXCL9 levels post neoadjuvant chemotherapy (Fig. 6D), indirectly supporting the contribution of TI-NK cells in promoting a CCL5/IFNɣ-dependent immunoreactive microenvironment.

## Discussion

The quantity of tumor-infiltrating lymphocytes (TIL score) is a prognostic factor for improved patient survival in triple-negative and HER2-positive breast tumor subtypes, and a predictor of response to anti-HER2 therapeutic antibodies in HER2-positive tumors [7, 26, 27]. However, the association of TI-NK cells with the efficacy of neoadjuvant treatment with anti-HER2 antibodies was largely superior to that of the TIL score, even though their numbers were relatively low. In this study, we showed that TI-NK cells act as major contributors to early CCL5/IFN-ɣ production along anti-HER2 antibody treatment in primary breast cancer patients, disclosing the role of the IFN-ɣ/CCL5/CXCL9 axis in the effectiveness of neoadjuvant treatment with anti-HER2 antibodies. In addition, our results further support anti-tumor immunity as a major mechanism of action of anti-HER2 antibodies in some patients, backing the potential of TI- NK cells or TI-NK cell surrogates as biomarkers of response in primary HER2-positive  breast cancer patients.

Mechanistic studies in in vitro and in vivo ADCC models indicate the importance of NK cells for the initial production of CCL5/IFN-ɣ, subsequently resulting in CXCL9/10 production by bystander breast tumor cells, during anti-HER2 antibody treatment. While the dependency on IFN-ɣ for CXCL9/10 induction was previously known [28], our data show the cooperation between IFN-ɣ and TNF-α in promoting the secretion of CCL5 and CXCL9 from breast cancer cells. Additionally, our findings reveal the dual effect of type I IFNs, which cooperated with IFN-ɣ and TNF-α for boosting CCL5 and CXCL10 secretion, yet negatively regulated CXCL9 production. Hence, these results indicate that dysregulated expression of CCL5, CXCL9 and CXCL10 may occur in specific contexts. It is likely that presence of TI-NK cells in primary tumors allows for the coordinated production of CCL5/IFN-ɣ/CXCL9, having an anti-tumoral effect by their paracrine action on immune cells [29]. On the other hand, excessive production of CCL5 and CXCL10 in the absence of IFN-ɣ and CXCL9 in metastatic lesions or primary tumors, refractory to immune infiltration [30], could have an autocrine pro-tumoral role [31–34]. Of note, inhibition of CXCL9/10 production by epigenetic silencing or soluble inhibitors, has been shown to facilitate tumor development in cancer models and patients [19, 35], supporting the importance of CXCL9/10 paracrine effect on immune cell recruitment. The putative relevance of these immunoevasion mechanisms along primary or acquired resistance to anti-HER2 antibody-based neoadjuvant treatment deserves attention.

Our study also indicates that two tumor-infiltrating NK cell subsets, CD16^+^ and CD16^-^ CD103^+^ , could be the initial suppliers of CCL5 and IFNɣ during chemotherapy and anti-HER2 antibody treatment in human breast tumors. Data from the in vivo model as well as from CD16+ and CD103^+^ NK cells in human breast tumors support their lineage relationship and provide evidence of their sequential conversion along activation within the tumor microenvironment. A conversion likely due to CD16 shedding upon NK cell activation and the presence of TGF-β which up-regulates their tissue-residency transcriptional program [36–39]. Of interest, the CD16^-^ CD103^+^ TI-NK cell subpopulation showed the highest expression of *IFNG*, *CCL5*, *XCL1* transcripts. These molecules have been previously related to NK cell activation in solid tumors and are required for the recruitment/survival of dendritic cells with the capacity to stimulate cytotoxic tumor-specific T cells [5, 6]. This finding aligns with the biological pathways enriched in tumor biopsies containing NK-cells. Moreover, recent studies have linked the presence of CD16^-^ NK cells with tissue residency traits, resembling CD16^-^ CD103^+^ , to the efficacy of PD1 blockade through the production of CCL5 [40].

On the other hand, a subpopulation of CD16^-^ CD103^-^ cells with a transcriptomic profile reminiscent of ILC3 was also included in the NK cell gate in human tumors, in accordance with expression of CD56 by ILC3 [41]. This subpopulation was negatively associated with overall NK cell proportions and correlated with immune contextures including high CD4/CD8 T cell ratios. Although the pro- or anti-tumoral role of ILC3 appears to be tumor/model-dependent, a CD56^+^ /ILC3 subset has been identified in tumor-draining lymph nodes and tumor samples from breast cancer patients with advanced disease [12, 42].

In addition to *CCL5/IFNG* expression in the diagnostic tumor biopsy our study also discloses the potential role of serum CCL5 and CXCL9 as early biomarkers of response to neoadjuvant treatment with anti-HER2 antibodies. Their early and coordinated increase upon treatment initiation occurred in patients with NK cell-infiltrated tumors and could indicate NK cell activation in response to chemotherapy-induced changes in tumor cells [43]. Indeed, plasma levels of IFN-ɣ-inducible chemokines CXCL9 and CXCL10 have been correlated with survival and chemotherapeutic efficacy in advanced ductal adenocarcinoma [44].

Overall, specialized TI-NK cell subsets contribute to the efficacy of anti-HER2 antibodies in primary breast cancer patients by triggering an early and coordinated production of CCL5/IFN-ɣ-CXCL9/10 chemokine axis which facilitates the subsequent recruitment /differentiation of adaptive anti-tumor immunity. The similarity between the immune composition of different tumor molecular subtypes supports the putative contribution of TI-NK cells to the clinical efficacy of treatments such as chemotherapy and immunotherapy in triple negative breast cancer or to the recently approved antibody–drug conjugates for HER2-positive tumors. Of note, Trastuzumab-deruxtecan, an antibody–drug conjugate with unprecedent efficacy in advance disease patients (DX-8951 derivative, DXd) [45, 46] has been shown to enhance anti-tumor immunity by promoting CXCL9/10 production by targeted tumor cells, increasing the number of tumor-infiltrating CD8^+^ T cells in preclinical models [47]. Further studies are warranted for analysing the potential value of CCL5, CXCL9 and IFN-ɣ as biomarkers of response to trastuzumab-deruxtecan and the possible synergism between trastuzumab-deruxtecan and tumor-infiltrating NK cells.

## Conclusion

This study shows that Natural Killer cells that infiltrate the tumor (TI-NK) produce CCL5 and IFN-ɣ upon activation, triggering the development of an effective anti-tumor immune response along anti-HER2 antibody-based neoadjuvant treatment. Our observations point to anti-tumor immunity as a major mechanism of action of anti-HER2 antibodies in primary HER2-positive breast cancer patients with NK cell-rich tumors and provide the rationale for investigating combinatorial approaches with immunotherapy in those patients. In addition, our study supports the potential of TI-NK cells or TI-NK cell surrogates as biomarkers for optimizing patient stratification and personalized clinical management.

### Supplementary Information

**Additional file 1.** Supplementary methods. **Supplementary Figure 1.** Clinicopathological features and DEG in NK cell rich virus NK cell desert HER2+ tumors. **Supplementary Figure 2.** Contribution of IFN-ɣ, TNF-α and type I IFNs to the production of CXCL9 and CXCL10 upon NK cell-medicated ADCC against SKBR3 cells. **Supplementary Table 1.** Clinicopathological features of tumor specimens included inflow cytometry analysis of immune in filtrates. **Supplementary Figure 3.** Identification of TI-NK cell and T cell subsets in treatment naïve breast carcinomas. **Supplementary Figure 4.** Influence of tumor subtype in the composition of immune infiltrates. **Supplementary Figure 5.** Gating strategy and differential expressed genes of circulating and tumor-infiltrating NK cell subsets. **Supplementary Table 2.** Clinipathological features of patients included in longitudinal chemokine studies. **Supplementary Figure 6.** Systemic CCL5, CXCL9 and CXCL0 in HER2+ breast cancer patients.

## Data Availability

Microarray and RNAseq data are available in a public, open access repository, under accession number GSE230540. Remaining data can be obtained upon reasonable request.

## Abbreviations

TI-NK: Tumor-infiltrating Natural Killer cells
NK: Natural Killer
IFN-ɣ: Interferon gamma
pCR: Pathological complete response
ADCC: Antibody-dependent cell cytotoxicity
ILC: Innate lymphoid cell
PBMC: Peripheral blood mononuclear cells
FFPE: Formalin-fixed paraffin-embeded
TNF-α: Tumor necrosis factor Alpha
rIFN-β: Recombinant interferon beta
IHC: Immunohistochemistry
Tz: Trastuzumab
TIL: Tumor infiltrating lymphocytes
DEG: Differentially expressed genes
NSG: NOD/Scid/γc-/- mice
TNBC: Triple Negative Breast Cancer
GSEA: Gene set enrichment analysis
T-Dxd: Trastuzumab-deruxtecan
