# Supplementary material for: NK cell-triggered CCL5/IFNγ-CXCL9/10 axis underlies the clinical efficacy of neoadjuvant anti-HER2 antibodies in breast cancer
Source: J Exp Clin Cancer Res. 2024 Jan 3;43:10. doi: 10.1186/s13046-023-02918-4 (PMC10763072; doi:10.1186/s13046-023-02918-4)

**SUPPLEMENTARY INFORMATION**

**SUPPLEMENTARY METHODS**

**Gene expression microarray analysis of FFPE breast tumor biopsies.**

Total RNA was isolated (RNeasy® Micro Kit, Qiagen) from 3 sequential, 10 µm thick, FFPE breast tumor sections from six TI-NK cell positive and six TI-NK cell negative biopsies. Amplification, labelling, and hybridization were performed according to the protocol GeneChip WT PLUS Reagent kit (P/N 703174 2017) and hybridized to the Human Clariom S Array (Thermo Fisher Scientific). For analysis, R programming (Version 3.4.3) Bioconductor and the Comprehensive R Archive Network (CRAN 2017) packages were used. Samples were background corrected, quantile-normalized and summarized to a gene-level using the robust multi-chip average (RMA). An empirical Bayes moderated t-statistics model (LIMMA) adjusted by Estrogen Receptor status was built to detect differentially expressed genes with a p-value<0.05 and a fold-change>1.5. Data represented in heatmaps was scaled by genes using the z-score normalization. Genes differentially expressed between TI-NK cell positive and negative biopsies were loaded into the Ingenuity Pathway Analysis (IPA) software to analyse the biological pathways and networks modulated in TI-NK cell positive versus TI-NK cell negative tumors.

**Cell lines and *in vitro* ADCC assays with purified NK cells.**

Human HER2-positive breast cancer cell lines SKBR3 and BT474 were grown in complete DMEM/F-12 medium (D6421, Sigma-Aldrich) supplemented with L-glutamine (0.5 mM, 25030-081, Gibco), penicillin/streptomycin (100 U/ml and 100 μg/ml, respectively, Gibco), sodium pyruvate (1 mM, Gibco), 10% FBS (10270106, Gibco) and HCC1954 cells were grown in complete RPMI 1640 Glutamax medium (72400-021, Gibco). Purified NK cells were cultured in the presence of breast cancer cells at 1:1 effector:target (E:T) ratio and trastuzumab (210ng/ml) for 24 hours. At the end of culture, cell-free culture supernatants were collected for cytokine/chemokine determination. Anti-IFNAR mAb (5 µg/ml, 407296, Calbiochem), anti-IFNɣ mAb (5 µg/ml, 14-7318-81, Invitrogen), anti-TNF-α mAb (infliximab, 50 μg/ml, Remicade®, Janssen) and their combinations were added along ADCC assays in blocking/neutralization experiments.

**Human NK cell expansion for *in vivo* ADCC model**

PBMC were cocultured with irradiated (40Gy) 8866 B-LCL feeder cells at 3:1 ratio for 10-12 days. On day 12 cells were cryopreserved until use. Prior to intratumoral injection., expanded cells were thawed and cultured overnight in complete RPMI supplemented with recombinant IL-2 (200 U/ml) prior to NK cell isolation by negative selection.

**RT-qPCR and flow cytometry analysis from *in vivo* treated xenografted tumors**

After completing three treatment cycles mice were sacrificed and the remaining tumors were collected and digested. Half of the obtained cell suspensions were used for RNA extraction using the RNAeasy kit (Quiagen). 500ng of RNA was retrotranscribed using SuperscriptIII (18080044, Invitrogen), according to the manufacturer’s instructions. Gene expression was analysed using the SYBR green gene expression assay (LightCycler 480 SYBR Green I Master, 04887352001, Roche). The following primers were used for human genes (5’ to 3’, forward; reverse): *IFNG* (TGTCCACGCAAAGCAATACA, ACTCCTTTTTCGCTTCCCTGT); *CCL5* (GCCTGTTTCTGCTTGCTCTT, AACTGCTGCTGTGTGGTAGA); *CXCL9* (TTCCTGCATCAGCACCAACC, TTTCTCGCAGGAAGGGCTTC); *CXCL10* (AACCTCCAGTCTCAGCACCA, TGCAGGTACAGCGTA). The relative gene expression was normalized to *B2M* (TTAGCTGTGCTCGCGCTACTCT, TGGTTCACACGGCAGGCATACT). qPCR reactions were performed on the Lightcycler 480 Real-Time PCR System (Roche Diagnostics) and analysed using Lightcycler 480 SW 1.5.1 at the Genomics Core Facility, Universitat Pompeu Fabra, Barcelona. The reaction included a 30 seconds (sec) preincubation at 40ºC, followed by 40 amplification cycles (10 sec at 95ºC, 20 sec at 60ºC and 10 sec at 72ºC). The second half of the obtained cells were stained with directly labeled anti-hCD45, anti-hCD56, anti-hCD3, anti-hCD16 and anti-hCD103 antibodies and analysed by flow cytometry on a BD LSR Fortessa or BD-LSRII (BD Bioscience) and analyzed with FlowJo software (v10.0.7, Tree Star).

**viSNE characterisation of breast tumor immune lymphocyte subsets**

vi-SNE was implemented for the analysis of major lymphocyte subsets (Amir et al, Nature Biotechnology, 2013, 31:545-552). Raw flow cytometry data was imported into R using flowCore and openCyto packages. A compensation matrix generated in FlowJo (v10.0.7, Tree Star) was exported an applied into R. Lymphocytes were gated based on forward and side scatter within the CD45+ alive (DAPI-) gate. Data from either 10,000 gated lymphocytes or 2,000-5,000 NK cells were concatenated. Barnes-Hut t-SNE was conducted using the Rtsne package and subset identification by Phenograph. Graphics were produced using the ggplot2 and RColorBrewer packages.

**Bulk RNAseq analysis of tumor-infiltrating NK cell subsets and**

Total RNA was isolated from each sorted NK cell subset using RNeasy® Micro Kit (74004, Qiagen) RNA samples were subsequently subjected to RNA-sequencing (RNA-seq) at the Genomics UPF facility at Universitat Pompeu Fabra, Barcelona. NGS libraries of each were prepared using the NebNext Ultra II Directional RNA Library Prep Kit for Illumina (New England BioLabs) with PolyA capture. The quality and concentration of the libraries were validated with TapeStation D1000 (Agilent Technologies). Libraries were pooled in equimolar proportions, amplified by qPCR with specific primers and sequenced in a NextSeq High output 2x75 cycles run (Illumina). Reads were mapped against the human reference genome GRCh38 using the GRCh38-89 Ensembl annotation with STAR software (version 2.7.9a). Counts were obtained with the HTSeq software and were normalized with log2 counts per million (CPM) with the weighted “trimmed mean method” (TMM) implemented in the edgeR package (version 3.14.0) with a prior.count=0.5. Lowly-expressed genes were filtered out (mean log2(CPM)<-2). Differential expression analyses were performed using the limma package with the voom transformation. Patient and surrogate variables were included in the model. Differentially expressed genes between the three TI-NK cell populations were established using a threshold of p-value<0,05 and |log2(FC)|>1. Pre-ranked gene set enrichment analysis (GSEA) was performed with GSEA (version 4.1.0) using the log(p value)*signFC. Gene sets tested for enrichment were obtained from the publicly available data from Koues et al. (Koues et al, Cell, 2016, 165:1134-1146) (GSE78897) by performing differential expression between CD56dim NK/ILC1/ILC3 using a threshold of adjusted p-value >0.005 and |log2(FC)|>1.

**SUPPLEMENTARY FIGURES AND TABLES**

**
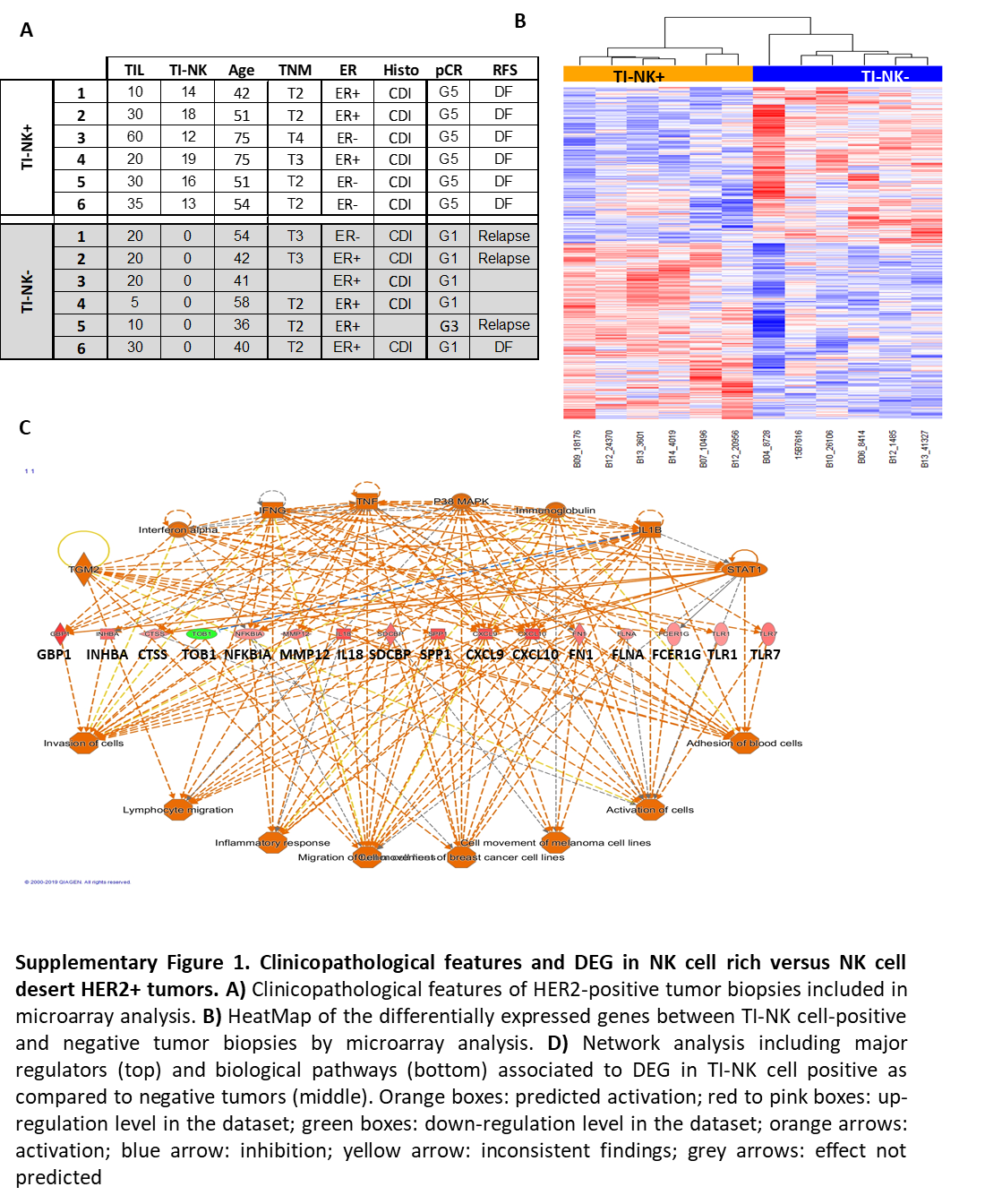
**


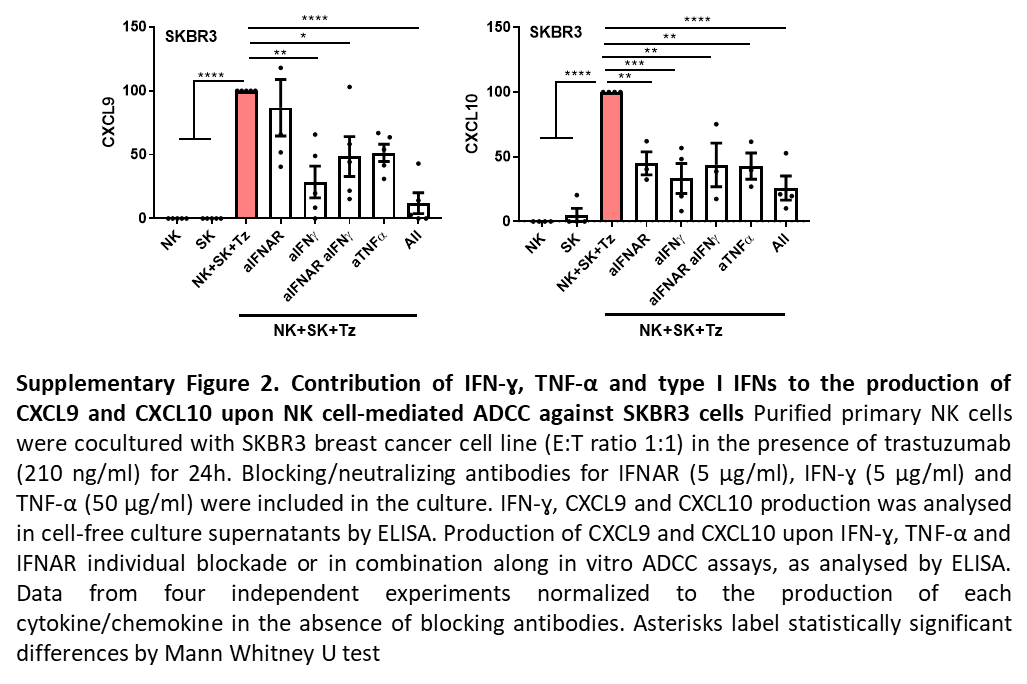


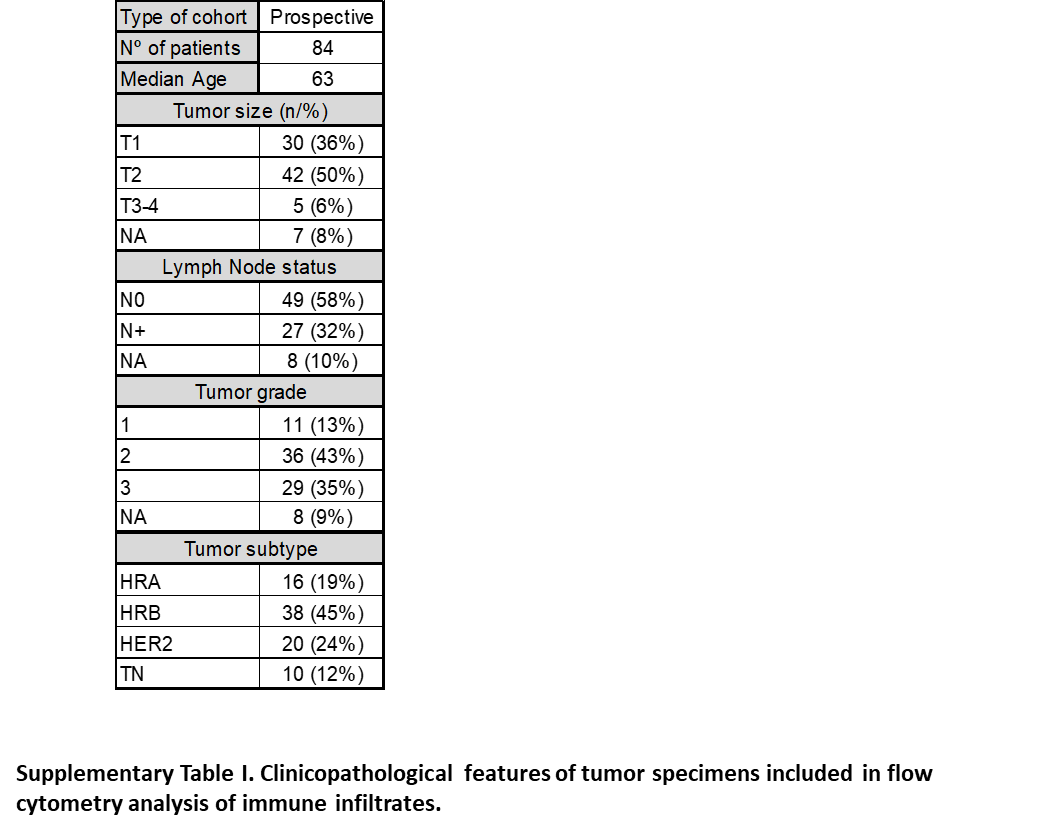


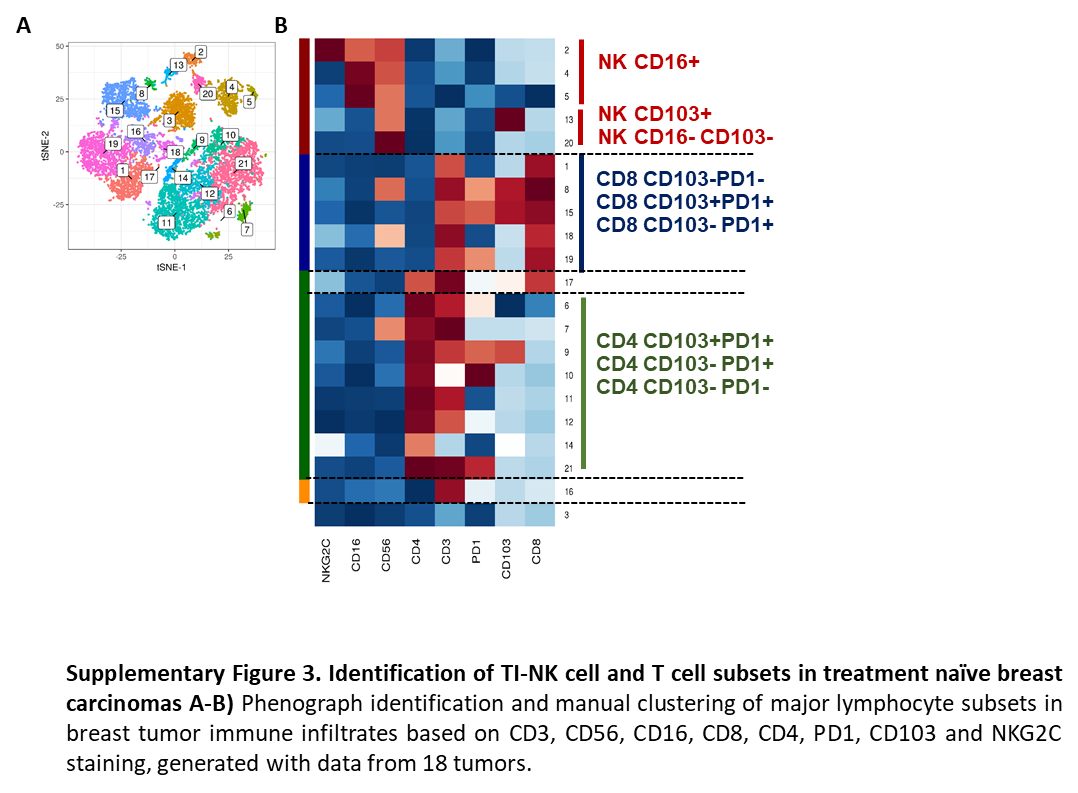


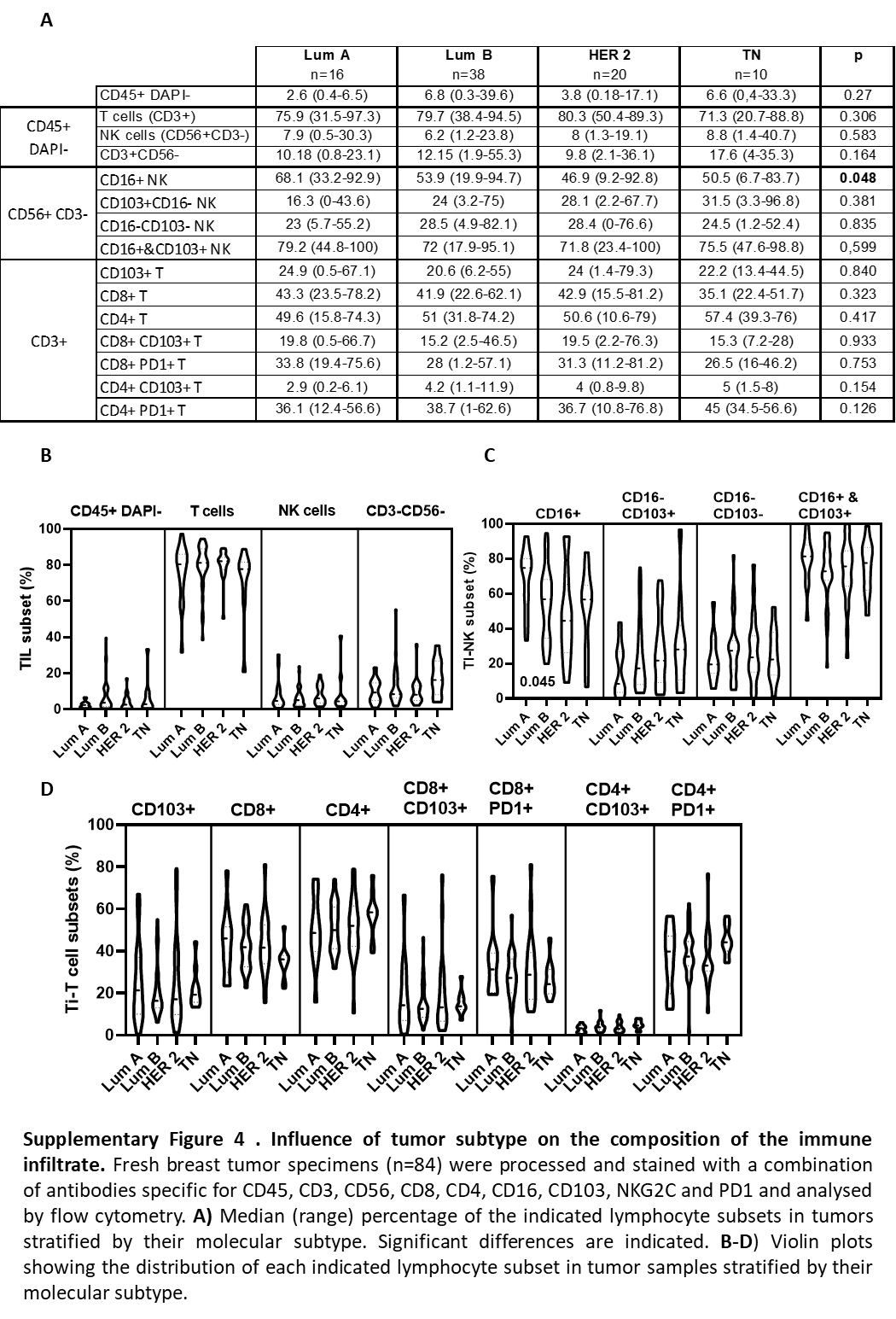


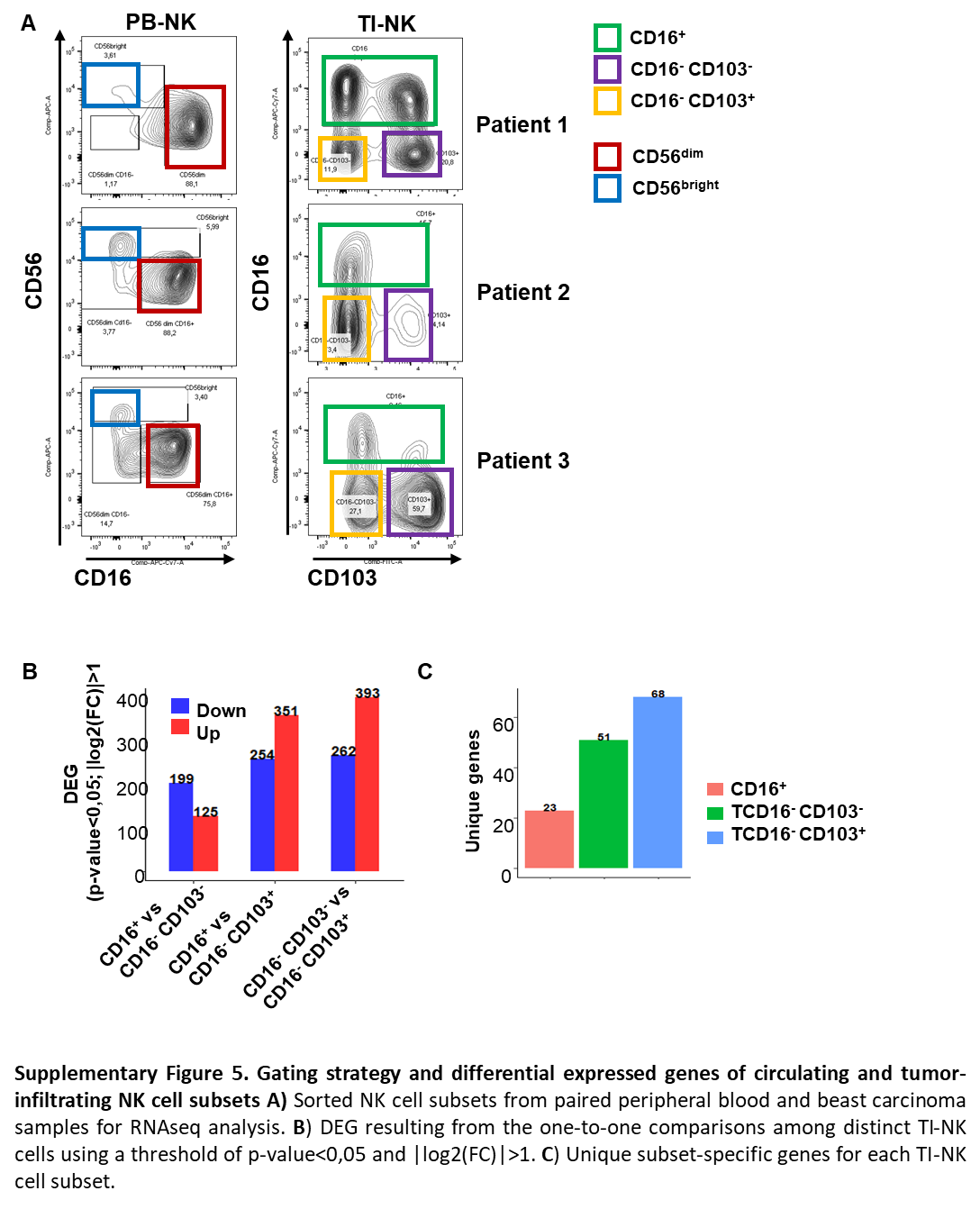


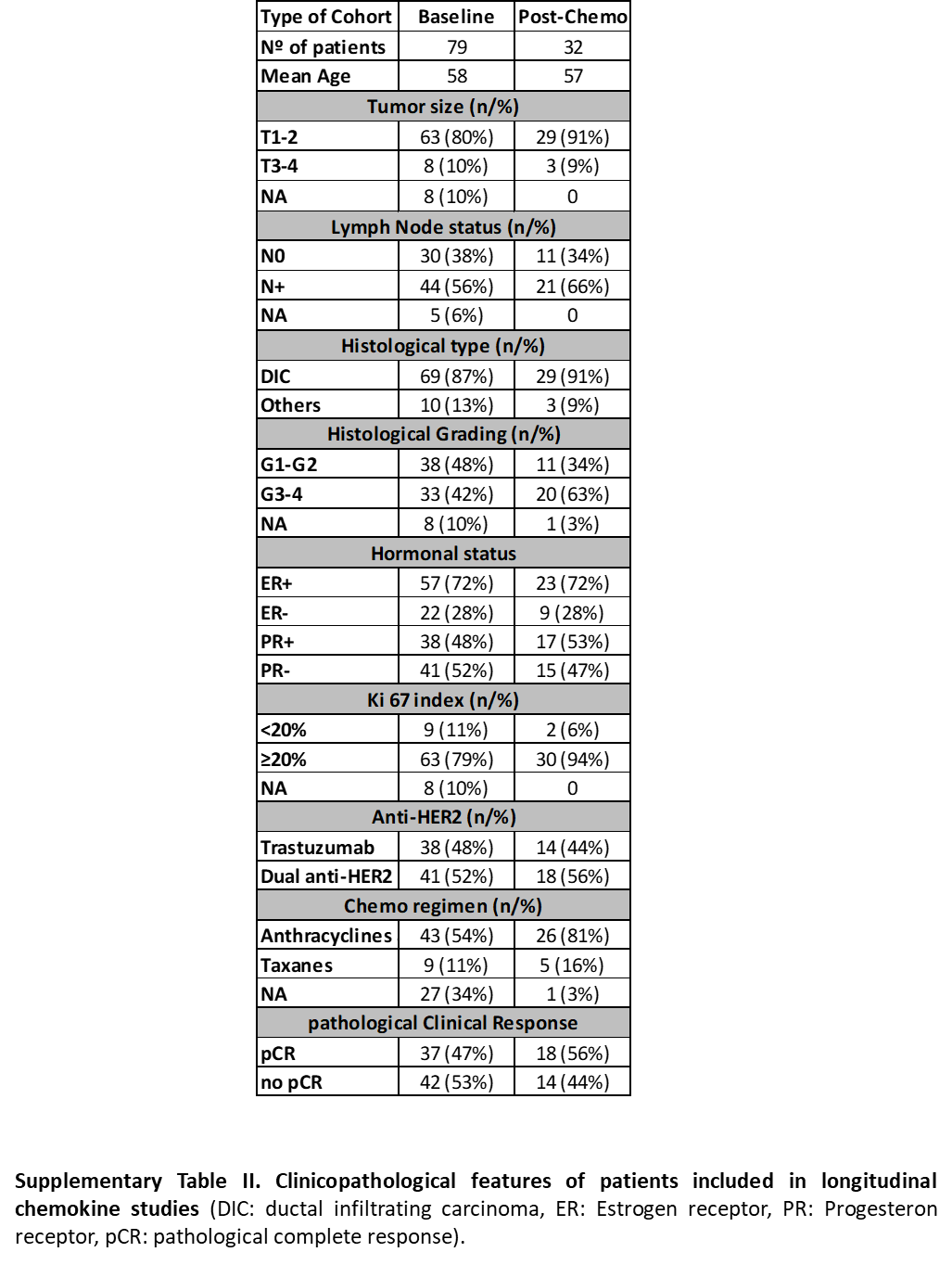


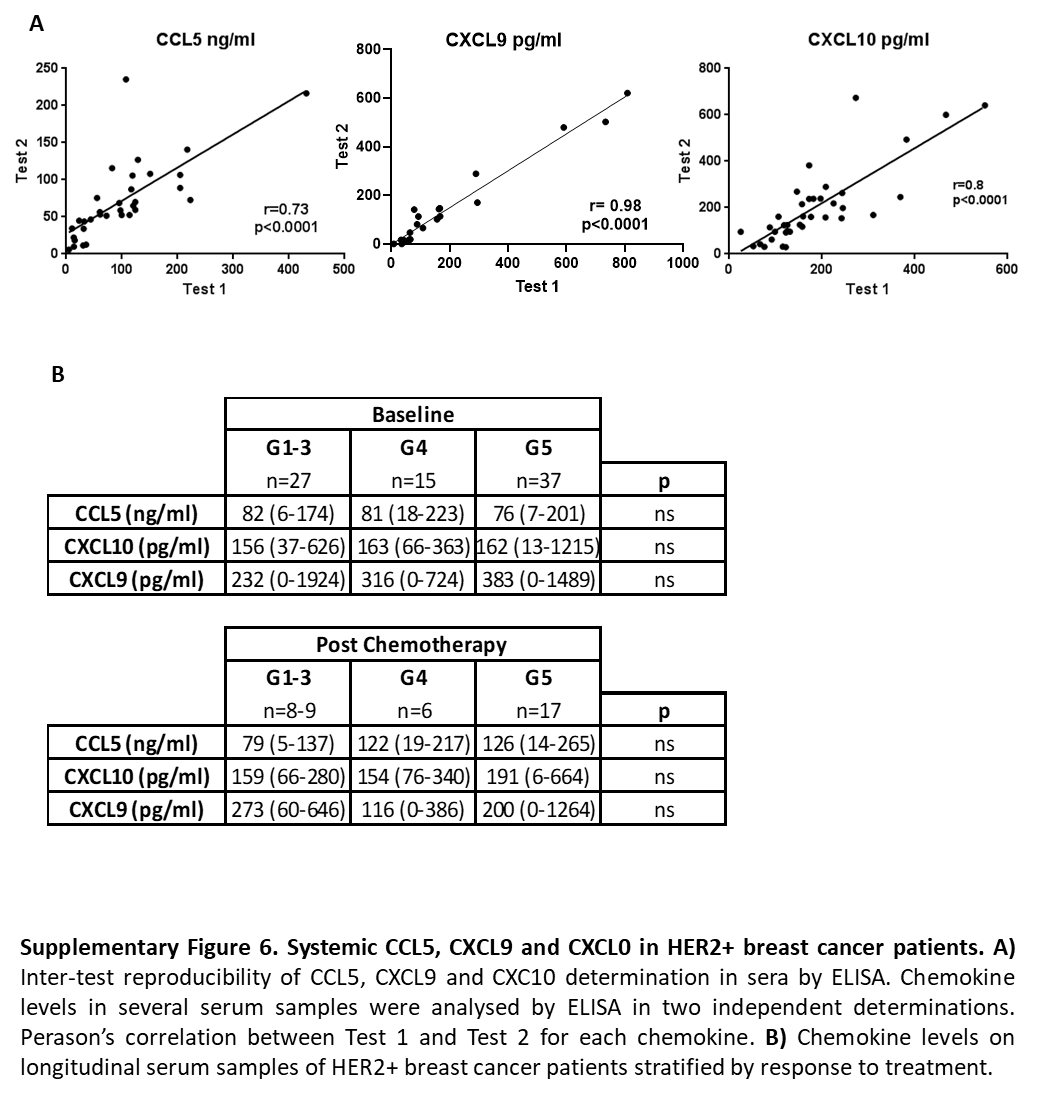

Supplement: Supplementary file 1 — Additional file 1. Supplementary methods. Supplementary Figure 1. Clinicopathological features and DEG in NK cell rich virus NK cell desert HER2+ tumors. Supplementary Figure 2. Contribution of IFN-ɣ, TNF-α and type I IFNs to the production of CXCL9 and CXCL10 upon NK cell-medicated ADCC against SKBR3 cells. Supplementary Table 1. Clinicopathological features of tumor specimens included inflow cytometry analysis of immune in filtrates. Supplementary Figure 3. Identification of TI-NK cell and T cell subsets in treatment naïve breast carcinomas. Supplementary Figure 4. Influence of tumor subtype in the composition of immune infiltrates. Supplementary Figure 5. Gating strategy and differential expressed genes of circulating and tumor-infiltrating NK cell subsets. Supplementary Table 2. Clinipathological features of patients included in longitudinal chemokine studies. Supplementary Figure 6. Systemic CCL5, CXCL9 and CXCL0 in HER2+ breast cancer patients. [file 13046_2023_2918_MOESM1_ESM.docx]
